# Transcranial pulse stimulation modulates spectral signatures of Alzheimer’s disease in the 3×Tg-AD mouse model

**DOI:** 10.1186/s13195-026-02109-1

**Published:** 2026-06-16

**Authors:** Irmak Gezginer, Maria Eleni Karakatsani, Prakruti Nanda, Pranati Chalasani, Diana Kindler, Rafael Storz, Markus Belau, Ruiqing Ni, Gerhard Schratt, Xosé Luís Deán-Ben, Daniel Razansky

**Affiliations:** 1 Institute for Biomedical Engineering, Institute of Pharmacology and Toxicology, Faculty of Medicine, University of Zurich, Zurich, Switzerland; 2 Institute for Biomedical Engineering, Department of Information Technology and Electrical Engineering, ETH Zurich, Zurich, Switzerland; 3 Laboratory of Systems Neuroscience, Institute for Neuroscience, Department of Health Science and Technology, ETH Zürich, Zurich, Switzerland; 4 Storz Medical AG, Tägerwilen, Switzerland; 5 Department of Nuclear Medicine, Inselspital University of Bern, Bern, Switzerland

**Keywords:** Alzheimer’s disease, Transcranial pulse stimulation, Resting-state functional magnetic resonance imaging, Fractional amplitude of low-frequency fluctuations

## Abstract

**Background:**

Large-scale brain network dysfunction is increasingly recognized as an important feature of Alzheimer’s disease (AD), offering insight into disease mechanisms and opportunities for targeted therapeutic intervention. The spectral features of this dysfunction remain poorly understood, and how neuromodulatory interventions interact with and reshape these frequency-resolved network signatures has yet to be explored.

**Methods:**

Triple-transgenic (3×Tg-AD) mice underwent resting-state functional MRI to assess functional connectivity, signal power, and variance across frequency bands after acute and longitudinal transcranial pulse stimulation (TPS), a low-intensity single-pulse neuromodulatory intervention. Novel object recognition testing was used to evaluate exploratory drive and short-term recognition memory following repeated TPS or sham treatment.

**Results:**

AD mice exhibited widespread functional connectivity loss accompanied by reduced low-frequency resting-state power and variance, together with a redistribution of spectral energy from slow-5 (0.01–0.027 Hz) to slow-4 (0.027–0.073 Hz) activity. TPS modulated these abnormalities by increasing low-frequency power, rebalancing slow-5/slow-4 fractional power, and strengthening network coherence, with the most prominent effects in cingulate, insular, piriform, and striatal regions. TPS effects showed a non-linear, region-dependent emergence across stimulation trains, with the strongest and most consistent modulation appearing after repeated stimulation. Similar spectral rebalancing was observed both after acute and longitudinal stimulation, persisting for up to 5 days. In addition, hippocampal regions that showed minimal acute responses exhibited delayed spectral changes at 24 h, with further modulation at 120 h. TPS-treated 3×Tg-AD mice did not show the decline in object exploration observed in sham-treated animals and showed an exploration-adjusted increase in novel object preference.

**Conclusions:**

Frequency-specific neural dynamics are sensitive markers of AD-related dysfunction and may provide a useful framework for tracking disease-related network abnormalities. TPS selectively modulates low-frequency oscillatory activity and network coherence and is accompanied by preliminary behavioral changes, including preserved exploratory engagement and an exploration-adjusted increase in novel object preference in a separate behavioral cohort. This highlights the potential of combining neuromodulation with spectral network analysis to monitor disease-related network dysfunction and treatment-associated responses.

**Supplementary Information:**

The online version contains supplementary material available at 10.1186/s13195-026-02109-1.

## Background

Growing evidence suggests that widespread neural network dysfunction significantly contributes to progressive cognitive decline in Alzheimer’s disease (AD) beyond the effects of its hallmark amyloid-β (Aβ) and tau pathologies. Even at the earliest stages, subtle disruptions in functional connectivity (FC) have been detected, foreshadowing the more conspicuous subsequent structural and cognitive decline [1, 2]. Large-scale brain networks, notably the default mode network (DMN), show disrupted FC in AD brain often before the onset of symptoms, with patients further showing abnormal neural oscillation patterns [3–5]. Reduced FC within the DMN has further been shown to correlate with Aβ accumulation, directly linking a molecular hallmark of AD to circuit-level dysfunction [6].

At the microscale, Aβ and tau pathologies impair synaptic signalling and propagate along axonal projections [7–10]. Transgenic models have been developed to replicate this cascade. Specifically, mice bearing human AD mutations develop Aβ plaques, tau tangles, synaptic loss, and network hypo-synchrony closely resembling the human disease [11–13]. Alongside spatial disconnection, AD has also been shown to disturb the temporal dynamics of neural activity [14–16]. Human electroencephalography (EEG) has consistently revealed a shift from faster alpha-beta rhythms toward slower delta-theta power [17]. Resting-state functional magnetic resonance imaging (fMRI) has complemented these findings by showing diminished amplitude of low-frequency (< 0.1 Hz) blood-oxygen-level-dependent (BOLD) oscillations in DMN hubs [18–21]. Band-resolved analyses (slow-5, 0.01–0.027 Hz; slow-4, 0.027–0.073 Hz) further delineated frequency-specific network deficits [22, 23]. However, existing studies in transgenic models harboring Aβ and/or tau have largely limited their analyses to a few predefined networks, often centered around hippocampal or default-mode-like circuits [24, 25]. Moreover, these studies rarely assess frequency-specific effects across the resting-state BOLD spectrum, leaving a comprehensive, frequency-resolved map of AD-related network pathology largely unexplored. As a result, undetected critical alterations in underexamined circuits may limit our understanding of the full spatial extent of AD-related network dysfunction.

Moreover, with network disconnection increasingly being acknowledged as an early driver of AD symptoms, restoring circuit integrity has the potential to significantly enhance therapeutic results. Multiple neuronal stimulation approaches have been shown to modulate brain circuits. Conventional non-invasive approaches such as transcranial magnetic stimulation (TMS) and transcranial alternating-current stimulation (tACS) have been shown to influence cortical rhythms and show cognitive benefits in AD and mild cognitive impairment, but their effects remain largely confined to superficial cortical regions [26]. Sensory gamma entrainment (40 Hz light or sound) has similarly been shown to enhance local oscillatory power but lacks precision for deep brain circuits [27]. Recently, transcranial pulse stimulation (TPS) has overcome these limitations by delivering ultrashort acoustic impulses capable of penetrating the intact skull to target both superficial and deep brain structures [28, 29]. Preliminary clinical trials in AD patients have reported cognitive improvement and strengthened memory-network connectivity after TPS treatments, suggesting its potential to counteract network disconnection [30, 31]. However, the precise mechanisms by which TPS modulates neuronal circuits remain unclear, largely due to limited preclinical studies. A systematic, mechanistic investigation of TPS-induced changes at both connectivity and spectral domains in a model with combined Aβ and tau pathologies can therefore provide crucial insights into the underlying neuromodulatory effects.

In this work, we systematically examined disrupted neural connectivity and frequency-specific alterations in regional brain activity in the triple transgenic (3×Tg-AD) mouse model characterized by both Aβ and tau pathologies, specifically aiming to investigate the ability of TPS to reshape whole-brain spectral dynamics. Male and female 3×Tg-AD mice and age-matched wild-type (WT) controls underwent resting-state fMRI to assess both immediate and sustained effects of TPS. In a separate cohort of aged female mice, longitudinal novel object recognition testing was used to determine whether repeated TPS influences exploratory engagement and short-term recognition memory. This combined design produced a spectral “fingerprint” of AD-related dysfunction, providing a benchmark to evaluate TPS-induced alterations across acute and delayed time scales while probing their behavioral relevance. We hypothesized that TPS would modulate aberrant low-frequency activity in 3×Tg-AD mice, shifting BOLD-derived network metrics toward WT-like profiles and potentially influencing behavioral measures related to exploratory engagement and novel object preference. Demonstrating such alterations would support further evaluation of TPS as a candidate neuromodulatory approach and provide insight into how single-pulse acoustic stimulation interacts with AD-related circuit dysfunction.

## Methods

### Animal models

Adult 3×Tg-AD mice (B6;129-Tg(APPSwe, tauP301L)1Lfa Psen1tm1Mpm/Mmjax; *n* = 37, 50–78 weeks; 10 male, 27 female) and age-matched wild-type B6129SF2/J controls (The Jackson Laboratory; *n* = 28, 50–78 weeks; 4 male, 24 female) were used in this study.

For the acute TPS in vivo MRI experiments, 3×Tg-AD mice (*n* = 12, 50–55 weeks; 8 male, 4 female) and age-matched wild-type controls (*n* = 8, 50–55 weeks; 4 male, 4 female) were used. For the longitudinal TPS experiments, a separate cohort of 3×Tg-AD mice was studied (*n* = 5, 66–68 weeks; 2 male, 3 female). Behavioral experiments were performed in aged female 3×Tg-AD mice (*n* = 20, 68–78 weeks) and age-matched female wild-type controls (*n* = 20, 68–78 weeks).

Animals were group-housed in individually ventilated cages under standard housing conditions (21–23 °C, 30–40% relative humidity) on a reversed 12-h light/dark cycle, with food and water available *ad libitum*. All experimental procedures were performed during the dark phase. 3×Tg-AD mice harbor human APP (Swedish), MAPT (P301L) and PSEN1 (M146V) mutations and exhibit amyloid and tau pathology; the B6129SF2/J line served as the background-matched control. All procedures were conducted in accordance with the Swiss Federal Act on Animal Protection and were approved by the Zurich Cantonal Veterinary Office (ZH079/22).

### In vivo MRI

Anesthesia was induced in wild-type B6129SF2/J (*n* = 8) and 3×Tg-AD (*n* = 17) mice with 5% isoflurane in air and maintained at < 1.5% during preparatory procedures. After positioning, animals received an intravenous bolus of medetomidine hydrochloride (0.05 mg kg⁻¹; Domitor, Pfizer, UK). Five minutes later isoflurane was lowered to 0.7% and a continuous infusion of medetomidine (0.1 mg kg⁻¹ h⁻¹) was initiated 10 min after the bolus. Functional MRI commenced 40 min after bolus delivery, following completion of a T2-weighted anatomical scan. Respiratory rate and rectal temperature were continuously monitored; body temperature was maintained at 37 °C with an MR-compatible feedback-controlled water blanket.

For the ramp-intensity TPS protocol, each imaging session comprised three consecutive 15-min resting-state runs. The first served as baseline (wild-type, *n* = 8; 3×Tg-AD, *n* = 8). For TPS experiments (3×Tg-AD, *n* = 6), mice were removed from the bore without disturbing head orientation and received the first low–low–high energy burst sequence (100 + 100 + 100 pulses; see TPS section). They were re-inserted, and the second fMRI run (AD-TPS1) began 44–71 s after stimulation. A second identical burst sequence (TPS2) was then applied, immediately followed by the third 15-min run (AD-TPS2).

For the fixed-intensity protocol (3×Tg-AD, *n* = 4), sessions began with a baseline scan, after which mice underwent sham handling: gel application and transducer placement for the same duration as real TPS stimulation but without acoustic pulse emission. 10-min resting-state scans were then interleaved with four medium-energy TPS trains (200 pulses each, see TPS section).

For the longitudinal stimulation protocol (3×Tg-AD, *n* = 5), animals first underwent a baseline resting-state fMRI scan, after which the first TPS session was administered. Following completion of six TPS sessions over 2 weeks (see TPS section), follow-up resting-state fMRI scans were acquired 24 h and 120 h after the final stimulation. Anesthesia was induced with 5% isoflurane in air and maintained at 2% during longitudinal stimulation sessions. Respiratory rate and rectal temperature were continuously monitored, and body temperature was maintained at 37 °C using a heating pad throughout the procedure.

### fMRI data acquisition

Imaging was performed on a 7-T small-animal scanner (BioSpec 70/16, Bruker BioSpin) equipped with a quadrature cryogenic surface coil (CryoProbe, Bruker BioSpin). Anatomical data were collected with a T2-weighted rapid acquisition with relaxation enhancement (RARE) sequence (field of view, 20 × 10 mm²; matrix, 256 × 128; 24 coronal slices; slice thickness, 0.4 mm; repetition time (TR), 3000 ms; echo time (TE), 15 ms; RARE factor, 8; 6 averages). Resting-state functional volumes were acquired using gradient-echo echo-planar imaging (GE-EPI) with the following parameters: field of view, 20 × 10 mm²; matrix, 90 × 50 (in-plane resolution, 0.22 × 0.20 mm²); 24 slices, 0.4-mm thickness; TR/TE, 1000/15 ms; flip angle, 60°; 1 average; 600–900 time points per run. Prior to functional runs, B₀ field maps were acquired to optimize local field homogeneity.

### fMRI data preprocessing

All functional data were processed in SPM12 (Wellcome Trust Centre for Neuroimaging), CONN v22a and in-house MATLAB (R2022b) scripts. For each animal, the T2-weighted anatomical volume was co-registered to its mean EPI and both images were subsequently normalized to the Allen Mouse Brain Common Coordinate Framework (CCF v3) [32] using sequential affine and nonlinear transformations. Functional volumes were resampled to 0.20-mm isotropic resolution, corrected for head motion by six-parameter rigid-body realignment, and spatially smoothed with a 0.6-mm full-width-at-half-maximum Gaussian kernel. Temporal preprocessing comprised linear detrending, despiking, and band-pass filtering (0.008–0.10 Hz) [33]. Nuisance regressors included mean white-matter and cerebrospinal-fluid signals together with the six motion parameters and their first derivatives [34, 35]; residual volumes were used for all subsequent connectivity and spectral analyses.

### Functional connectivity analysis

Denoised residual volumes were parcellated according to the Allen Mouse Brain Common Coordinate Framework (Supplementary Table 1). For each animal, the mean BOLD time series for every ROI was extracted and pairwise Pearson correlation coefficients were calculated. Correlation values were Fisher *z*-transformed prior to group-level statistics. Wild-type and 3×Tg-AD baseline matrices were compared with two-tailed Student t tests at each edge, followed by Benjamini–Hochberg false-discovery-rate correction (FDR < 0.05).

### Power spectral analysis

Regional BOLD time series were extracted from predefined ROIs as previously described. Power spectral density (PSD) was estimated using Welch’s method, applied to band-pass filtered signals (0.008–0.1 Hz) to characterize low-frequency oscillatory activity. The overall amplitude of the PSD, quantified as the amplitude of low-frequency fluctuations (ALFF), was computed by integrating the power spectrum across the full low-frequency range and taking the square root of the resulting value. The fractional ALFF (fALFF) was calculated as the ratio of power within a given frequency band to the total power across the entire frequency range. For group-level comparisons, PSD values were sampled at four evenly spaced frequencies within the 0.008–0.1 Hz range. In addition, the temporal variance of each time series was calculated to assess fluctuations in signal amplitude. Group comparisons of PSD, ALFF, fALFF, and variance were performed using paired two-tailed t-tests within the 3×Tg-AD group (e.g., pre- vs. post-stimulation) and unpaired two-tailed t-tests between 3×Tg-AD and WT groups. Fractional power was specifically assessed within the canonical slow-5 (0.01–0.027 Hz) and slow-4 (0.027–0.073 Hz) bands via fALFF to evaluate frequency-specific alterations. Effect sizes between groups were estimated using Cohen’s *d*.

### Independent component analysis (ICA)

Group-level ICA was performed in the CONN toolbox (v22a) to decompose low-frequency resting-state BOLD data into 15 spatially independent components. The analysis included combined datasets from 3×Tg-AD mice subjected to ramp-intensity stimulation and age-matched WT controls. For each component, the associated temporal time series was extracted and further analyzed. PSD and signal variance were computed for each time series to quantify oscillatory dynamics and signal stability. Group comparisons of PSD and variance were conducted using paired two-tailed t-tests within the 3×Tg-AD group (e.g., pre- vs. post-stimulation), and unpaired two-tailed t-tests for comparisons between 3×Tg-AD and WT groups.

### Transcranial Pulse Stimulation (TPS)

TPS was delivered using a CE-certified medical device (Neurolith, Storz Medical AG, Switzerland) that emits ~ 3 µs duration impulses. The probe was coupled to the intact scalp with degassed gel and oriented perpendicular to the skull so that the elongated focus (FWHM ~ 56 mm axial × ~5.3 mm lateral) coincided with the cortical surface; pulse repetition rate was 4 Hz. Under this clinical scale configuration, TPS generated an elongated acoustic field that covered a large portion of the murine brain, including midline and deeper structures, rather than a single tightly confined focal spot. During each stimulation session, a hand-held scan of the transducer was systematically performed across the skull in horizontal and vertical directions to provide broad whole-brain exposure.

Acoustic dosimetry at the applied energy flux density (EFD) of 0.05 mJ/mm² (PRF = 4 Hz) yields a free-water spatial-peak temporal-average intensity (I_SPTA_) of 16 mW/cm² and a spatial-peak pulse-average intensity (I_SPPA_) of 22 W/cm², with estimated intracranial values (after murine skull derating) of approximately 6.7 mW/cm² and 9.3 W/cm², respectively (see Supplementary Table 2 for a summary of acoustic descriptors). The estimated intracranial peak rarefactional pressure is ~ 1.4 MPa. These values lie 14-fold and 20-fold below the respective FDA diagnostic cephalic limits for I_SPTA_ (94 mW/cm²) and I_SPPA_ (190 W/cm²). Note that the mechanical index (MI), a cavitation-related safety metric derived for long duration sinusoidal waveforms used in diagnostic ultrasound, is not directly applicable to the broadband ultrashort (~ 3 µs duration) impulses used in TPS [36, 37]. TPS dosimetry is natively characterised by EFD, the parameter used for device certification (CE mark, 2018) and all published clinical TPS trials.

Two acute stimulation paradigms were applied. In the ramp-intensity protocol, mice received three bursts of 100 impulses at 0.05 mJ mm^− 2^, 0.05 mJ mm^− 2^ and 0.25 mJ mm^− 2^ (low-, low-, high-energy sequence; 300 impulses per TPS block; 60 s inter-burst interval), and the stimulation was repeated once after 15 min resting-state acquisition (total 2 TPS blocks and 600 impulses). In the fixed-intensity protocol, four blocks of 200 impulses were delivered at 0.10 mJ mm^− 2^, each block separated by a resting-state fMRI run, yielding 800 impulses per imaging session.

For each stimulation session in the longitudinal paradigm, mice received three bursts of 100 impulses at 0.10 mJ mm^− 2^ (300 impulses per session; 60 s inter-burst interval). A total of six identical stimulation sessions were delivered over 2 weeks. In the longitudinal fMRI cohort, the first TPS session was administered immediately after the baseline resting-state acquisition and follow-up resting-state fMRI scans were acquired 24 h and 120 h after the final TPS session. In a separate longitudinal behavioral cohort, behavioral assessment was performed before the first treatment session and immediately after the final treatment session.

### Behavioral experimental design

To assess behavioral outcomes following repeated TPS, aged female mice were used to model advanced AD-like pathology. Animals were assigned to one of four experimental groups (*n* = 10 per group): transgenic mice receiving TPS (3×Tg-AD + Stim), transgenic procedural controls (3×Tg-AD + Sham), wild-type mice receiving stimulation (WT + Stim), and wild-type procedural controls (WT + Sham). The longitudinal study design comprised a baseline behavioral assessment, a two-week TPS treatment period, and an immediate post-treatment behavioral assessment.

During the two-week treatment phase, mice in the active treatment groups (3×Tg-AD + Stim and WT + Stim) received a total of six TPS sessions over 2 weeks. To control for the potential confounding effects of anesthesia, handling, and immobilization, mice in the procedural control groups (3×Tg-AD + Sham and WT + Sham) underwent an identical protocol consisting of six sessions. Sham animals were anesthetized and positioned under the TPS transducer for the same duration as their respective Stim cohorts, but no acoustic stimulation was delivered.

### Novel object recognition (NOR) task

Short-term recognition memory was evaluated using the novel object recognition (NOR) task, conducted in an open-field arena (TSE Systems) over two consecutive days. Twenty-four hours before testing (Day 1), mice underwent a 10-minute habituation session to acclimate to the testing environment. On the testing day (Day 2), the protocol consisted of three sequential 5-minute phases. Mice first underwent an additional habituation phase in the empty arena (Hab 1). This was immediately followed by the familiarization phase (Hab 2), during which mice were allowed to freely explore two identical objects. After a 10-minute retention interval, during which the animals were temporarily housed in a previously acclimated cage, the test phase was administered. In the test phase, one of the familiar objects was replaced with a novel object, and the mice were allowed to explore for 5 min. To prevent sensitization or the development of object preference across the longitudinal study design, distinct, counterbalanced object sets were used for the baseline and post-treatment testing time points. All behavioral sessions were recorded using a 25-frame-per-second camera to enable precise tracking and quantification of rapid behaviors and transient object interactions.

### Behavioral analysis

The video recordings were manually analyzed by a trained investigator blinded to the experimental condition and animal genotype. Object interaction was defined as the animal’s snout being directed toward the object within its immediate vicinity, using a constant proximity threshold across all object sets and testing sessions, or as active physical investigation, such as sniffing or rearing to inspect the top of the object. Accidental contact, such as bumping into the object while walking or using the object as support while scanning the arena, was excluded. These defined interactions were summed to determine the time spent actively exploring the familiar and novel objects during the test phase.

To quantify recognition memory, a novel object preference index was calculated for both the baseline and post-treatment test sessions as:

$$\mathrm{Preference}\;\mathrm{Index}=\frac{\mathrm{Time}_{novel}-\mathrm{Time}_{familiar}}{\mathrm{Time}_{novel}+\mathrm{Time}_{familiar}}$$

Positive values indicate preferential exploration of the novel object, whereas values around zero indicate no preference between the novel and familiar objects. Animals with zero total object interaction during the test phase were excluded from the preference index analysis, since object discrimination could not be meaningfully quantified in the absence of active exploration.

Because the preference index may be influenced by overall exploratory drive, total object exploration time during the test phase was explicitly included as a covariate in the statistical model. This allowed us to assess whether changes in novel object preference reflected altered recognition memory rather than differences in the total amount of object-directed behavior.

Statistical analyses were performed in MATLAB using linear mixed-effects models implemented with *fitlme*. To directly evaluate treatment-related changes within each genotype, separate models were fitted for WT and 3×Tg-AD mice. In each genotype-specific model, the novel object preference index was modeled as a function of treatment condition, time, and their interaction, with total exploration time included as a continuous covariate. To account for repeated measurements across baseline and post-treatment sessions, a random intercept was included for each animal. The model structure was:

$$\text{Preference Index}\sim\:\mathrm{Treatment}\times\:\mathrm{Time}+\text{Total Exploration Time}+(1\mid\:\mathrm{Animal})$$

where treatment indicated Stim versus Sham, time indicated pre-treatment versus post-treatment, and total exploration time corresponded to the summed exploration of the novel and familiar objects during the test phase. Total exploration time was mean-centered before model fitting to improve interpretability of the model intercepts.

This genotype-specific modeling strategy was used to answer two main questions. First, within each genotype and treatment group, we tested whether novel object preference changed from baseline to post-treatment after controlling for total exploration time. Second, within each genotype, the treatment-by-time interaction was used to test whether the pre-to-post change differed between Stim and Sham animals. Thus, the analysis assessed whether TPS was associated with improved novel object discrimination while accounting for individual differences in exploratory behavior and repeated measurements within the same animals.

After model fitting, planned contrasts were used to estimate post-treatment versus pre-treatment changes in preference index within each genotype and treatment group. Additional planned contrasts assessed whether the pre-to-post change differed between Stim and Sham animals within each genotype. While animals with zero total object interaction were excluded to ensure the preference index reflected active discrimination, all other data were maintained in their raw form. This approach allowed for a robust evaluation of behavior as is, rather than enforcing interaction thresholds, typically derived from younger, healthier mice that may not account for this cohort’s pathologically burdened state.

### Immunohistochemistry and microscopy

Mice were anesthetized with ketamine (100 mg/kg), xylazine (10 mg/kg), and acepromazine maleate (2–3 mg/kg) (total volume 100 µL), then transcardially perfused, first with phosphate-buffered saline (PBS) and subsequently with 4% paraformaldehyde (PFA) in PBS. Extracted brains were post-fixed in 4% PFA for 4 h at 4 °C, followed by cryoprotection in 30% sucrose in PBS for 3 days. Specimens were embedded in optimal cutting temperature (OCT) compound and sectioned into coronal sections to a thickness of 35 μm using a cryostat (CM3050S, Leica Biosystems, Germany). 5–8 free-floating sections with a 6-sections gap were first washed in 0.1 M phosphate buffered saline (PBS) and blocked in a solution of 5% donkey serum in 0.3% PBS with Triton X-100 (PBST) for 30 min. The tissue was probed with mouse HT7, a tau monoclonal antibody (Product # MN1000, Invitrogen) at a dilution of 1:1000 at 4 °C. On the second day, sections were washed in 0.3% PBST and then incubated for 60 min in a solution of 5% donkey serum in 0.3% PBST containing donkey-raised secondary Alexa Fluor 488 antibody (1:1000, ab150105, Abcam). The tissue was then washed with 0.1 M PBS and mounted on slides. After allowing the tissue to dry, the slides were cover-slipped with Dako Fluorescence Mounting Medium (S302380-2, Agilent). Large field confocal images of the coronal slices were captured on a Zeiss LSM 900 AiryScan 2 confocal microscope (Carl Zeiss AG, Oberkochen, Germany). A 10x objective with 0.3 NA was employed, corresponding to 5.2 mm working distance and 1.1 μm resolution at the 520 nm excitation wavelength.

## Results

### 3×Tg-AD mice exhibit disrupted connections and frequency-specific shifts in regional brain activity

**Fig. 1:**
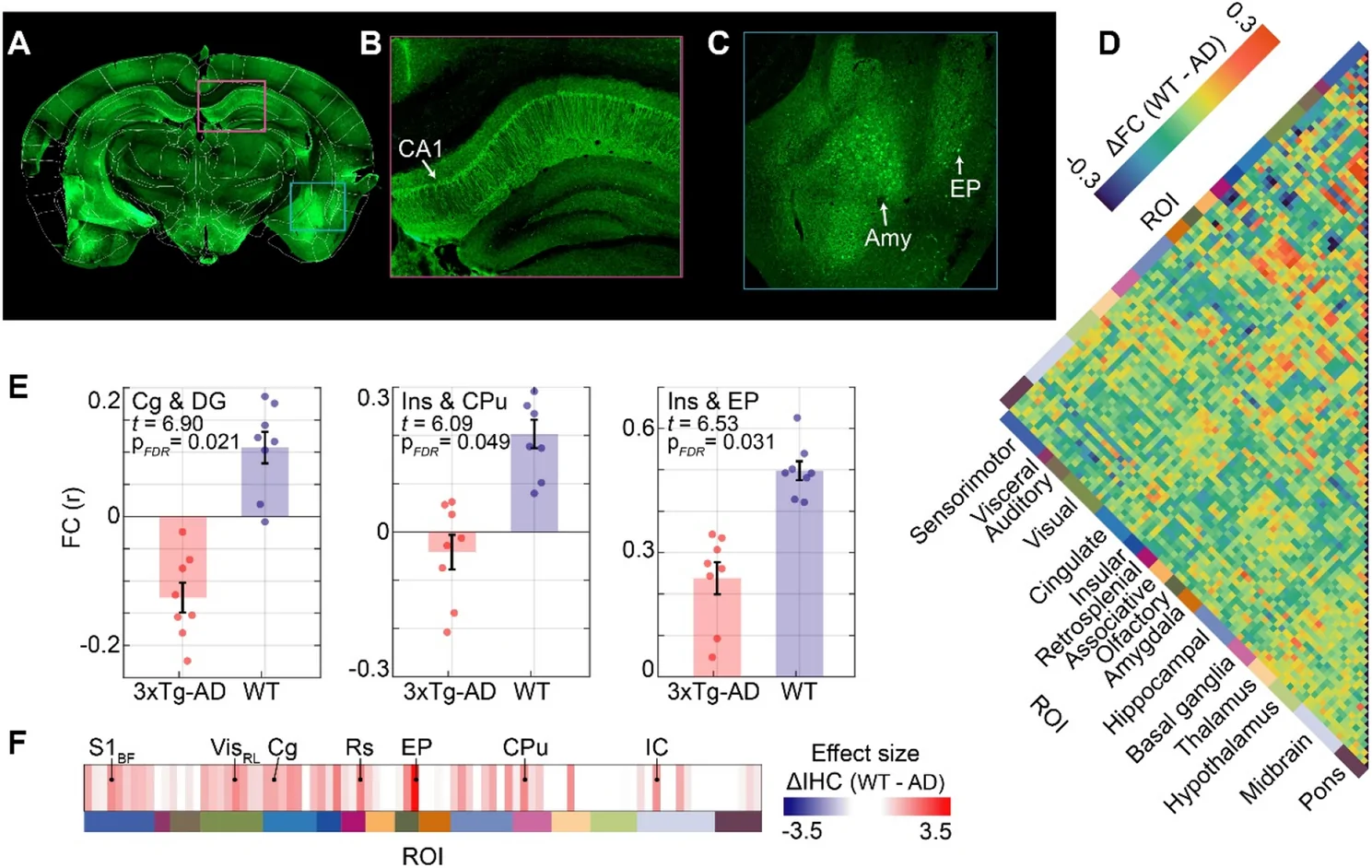
Tau accumulation parallels widespread reductions in resting-state functional connectivity (FC) in 3×Tg-AD mice.** A-C** Representative HT7 immunohistochemistry for human tau in a 52-week-old female 3×Tg-AD mouse, showing dense labelling in CA1 (B) and the amygdala/endopiriform nucleus (Amy/EP) (C). **D** Group-difference matrix (wild-type, WT, minus 3×Tg-AD; *n* = 8 per group) for whole-brain functional connectivity. Differences cluster in auditory, visual, cingulate, retrosplenial and hippocampal networks. **E** Weakened connections in 3×Tg-AD mice. FC is lower in 3×Tg-AD between anterior cingulate cortex (Cg) and dentate gyrus (DG), and between insula (Ins) and both caudoputamen (CPu) and EP. Data are shown as mean ± s.e.m.; two-tailed t-test, false-discovery-rate (FDR) < 0.05. **F** Effect size (Cohen’s *d*) map of interhemispheric connectivity (IHC) reveals widespread bilateral disconnection, encompassing somatosensory, visual, cingulate, insular, retrosplenial, piriform, hippocampal, striatal networks. S1_BF_, primary somatosensory cortex, barrel field; Vis_RL_, rostrolateral visual area; Rs, retrosplenial cortex; IC, inferior colliculus

The 3×Tg-AD mouse model is extensively used in AD research as it recapitulates hallmark pathological features observed in humans, including both Aβ plaques and tau tangles, thereby providing a valuable platform for studying disease progression and evaluating potential therapeutic interventions. HT7 immunostaining confirmed pronounced human tau deposition in the established 3×Tg‑AD mouse colony, extending beyond the hippocampus to the amygdala, piriform cortex, and hypothalamic nuclei (Fig. 1a–c). Resting-state fMRI revealed that this pathological burden translates into extensive disruptions in FC, with significant differences between age-matched (50–55 weeks-old) WT and 3×Tg-AD mice, clustering within auditory, visual, cingulate, and retrosplenial networks (Fig. 1d, SFig. 1). Consistent with earlier findings [5, 6, 38, 39], connectivity between the anterior cingulate cortex (a DMN hub) and dentate gyrus was reduced in 3×Tg-AD mice (FC_AD_ = -0.126 ± 0.023, FC_WT_ = 0.107 ± 0.025; t(14) = 6.90, p_FDR_ = 0.021). A similar decrease in FC was observed between the dentate gyrus and caudoputamen (SFig. 2). Beyond these previously reported hippocampal and DMN disruptions, we identified substantial FC impairments within less explored associative and subcortical pathways. Specifically, connectivity reductions were evident between insular cortex and both caudoputamen (FC_AD_ = -0.042 ± 0.036, FC_WT_ = 0.203 ± 0.029; t(14) = 6.09, p_FDR_ = 0.049) and endopiriform nucleus (FC_AD_ = 0.088 ± 0.025, FC_WT_ = 0.256 ± 0.017; t (14) = 6.53, p_FDR_ = 0.031) (Fig. 1e). Interhemispheric connections, another hallmark disrupted in AD [40–42], were significantly diminished in 3×Tg-AD animals, extending beyond the hippocampus to include endopiriform cortex, primary sensory and visual areas, cingulate and retrosplenial cortices, caudoputamen, and inferior colliculus (Fig. 1f, SFig. 3). Together, these findings point to a comprehensive network-level impairment in 3×Tg-AD mice, far exceeding traditionally studied circuits.

**Fig. 2:**
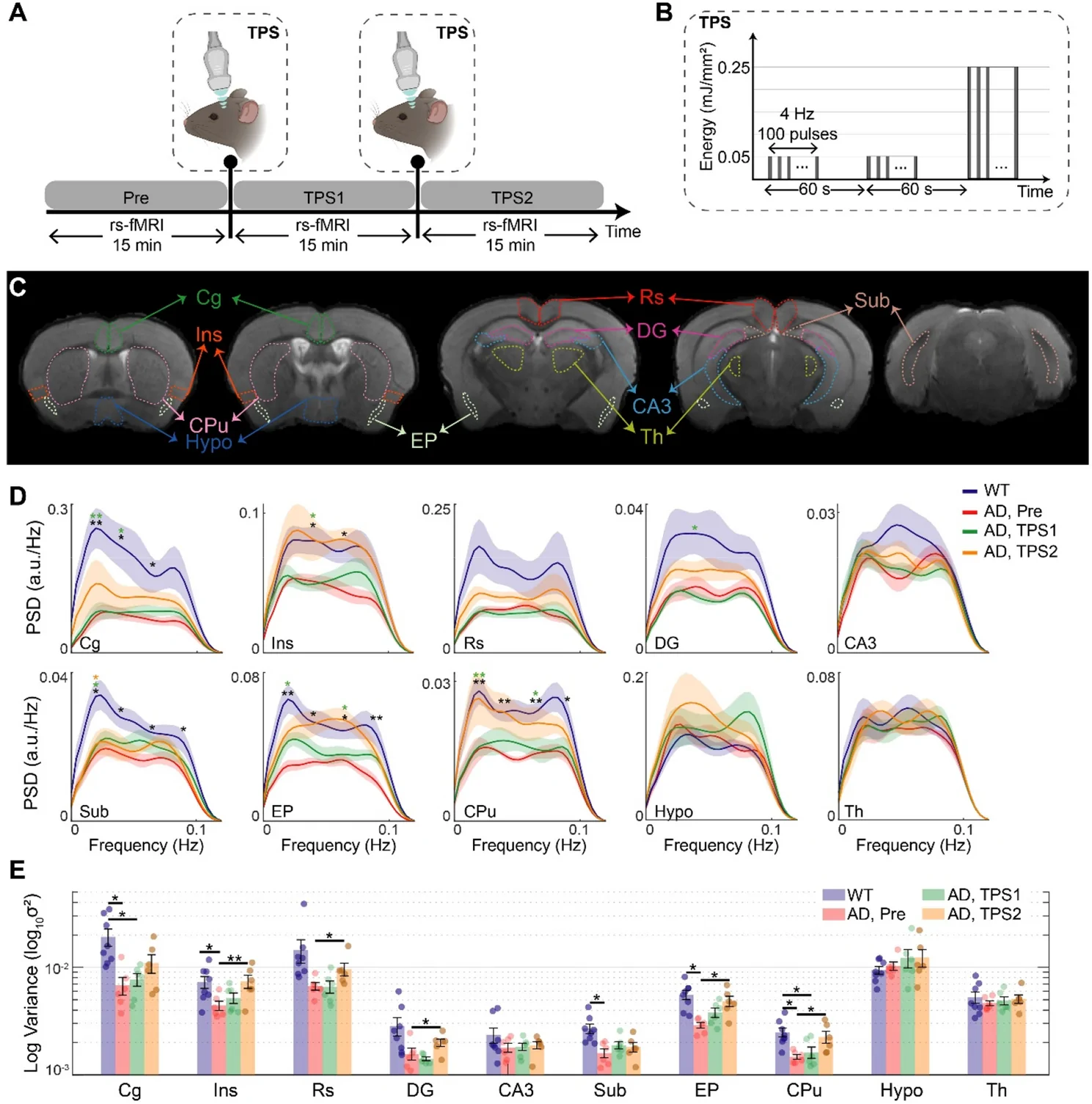
Transcranial pulse stimulation (TPS) modulates low-frequency BOLD oscillations. **A-B** Experimental design of the ramp-intensity TPS protocol. Three 15-min resting-state fMRI measurements (Pre, TPS1, TPS2) were separated by identical stimulation where mice received three 100-pulse bursts at 0.05 mJ mm^− 2^, 0.05 mJ mm^− 2^ and 0.25 mJ mm^− 2^ (low-, low-, high-energy sequence; 60 s inter-burst interval). **C** Anatomical locations of nine regions of interest (ROIs) overlaid on a T2-weighted anatomical image. **D** Power-spectral density (PSD) of the low-frequency BOLD band (0.008–0.10 Hz) for each ROI under four conditions: WT, 3×Tg-AD before stimulation (AD-Pre), after one TPS train (AD-TPS1) and after a second TPS train (AD-TPS2). Shaded areas, s.e.m. Stars denote frequency bins with significant group differences and are color-coded (black = WT vs. AD-Pre; green = WT vs. AD-TPS1; yellow = WT vs. AD-TPS2; unpaired t-test for WT vs. AD-Pre and paired two-sided t-test with FDR correction for within-AD comparisons; (*) *p* < 0.05, (**) *p* < 0.01). **E** Variance in the BOLD time series for the same ROIs. Bars, mean; error bars, s.e.m.; unpaired t-test (WT vs. AD-Pre) and paired two-sided t-test with FDR correction for within-AD comparisons; (*) *p* < 0.05, (**) *p* < 0.01, (***) *p* < 0.001. Cg, anterior cingulate area dorsal part; Ins, agranular insular area dorsal part; Rs, retrosplenial area dorsal part; DG, dentate gyrus; Sub, subiculum; EP, endopiriform nucleus; CPu, caudoputamen; Hypo, lateral hypothalamic area; Th, posterior complex of the thalamus. Hypo and Th served as control regions that showed no genotype-specific differences

To further characterize these connectivity alterations, we investigated their spectral underpinnings, an approach seldomly applied in animal models of AD. In humans, AD has been associated with characteristic frequency-domain abnormalities, notably redistribution of EEG oscillatory power toward slower frequency [43, 44]. To examine if similar temporal dynamic shifts occur in 3×Tg-AD mice, we conducted power-spectral density (PSD) analyses of low-frequency (< 0.1 Hz) BOLD signals across the whole brain. This revealed a distinct spectral signature in 3×Tg-AD animals (Fig. 2). Compared to WT controls, regional power was significantly lower in the anterior cingulate cortex, posterior insula, subiculum, endopiriform nucleus, and caudoputamen, while retrosplenial and hippocampal areas exhibited subtler, qualitative differences (Fig. 2). Other regions, e.g. thalamic and hypothalamic areas, remained largely unaffected (Fig. 2).

### TPS selectively modulates region-specific spectral abnormalities

To test the hypothesis that TPS can normalize the observed aberrant low-frequency oscillations, we applied repeated trains of TPS and monitored subsequent spectral changes in 3×Tg-AD mice (*n* = 6). Resting-state fMRI was acquired before and after two identical TPS sessions (Fig. 2a). We used a ramp-intensity design composed of low–low–high energy stimulation, previously shown to induce robust hemodynamic changes (Fig. 2b) [29, 37]. Following the second TPS session, PSD profiles in the insular cortex, endopiriform nucleus, and caudoputamen shifted toward WT-like levels (Fig. 2c, d). Anterior cingulate cortex power spectrum partially normalized, indicated by re-emergence of a WT-like spectral peak, although amplitude differences remained modestly present. Notably, hippocampal regions, including the subiculum, showed no significant PSD changes post-TPS (Fig. 2d). Signal variance, an independent measure of spontaneous activity fluctuations, was also lower at baseline in 3×Tg-AD mice across multiple regions (cingulate, insula, subiculum, endopiriform nucleus, caudoputamen; Fig. 2e). TPS significantly increased the variance in these regions (excluding subiculum) and also enhanced variance in retrosplenial cortex and dentate gyrus (Fig. 2e). Importantly, exploratory sex-stratified analyses of the acute cohort showed broadly similar TPS-associated trends in males and females, although subgroup sizes were too small for definitive inference (SFig. 4). In both sexes, some regions showed modulation already after the first stimulation, but the clearest and most consistent effects across TPS-responsive regions emerged after the second stimulation, suggesting a non-linear and region-dependent response profile that may depend on repeated stimulation and/or sufficient cumulative stimulation energy. It is important to note that the exploratory sex-stratified analyses were conducted to assess whether the main TPS-associated effects were grossly driven by one sex. Because subgroup sizes were small, these analyses were considered descriptive only and were not used to infer sex-specific TPS responsiveness.

**Fig. 3:**
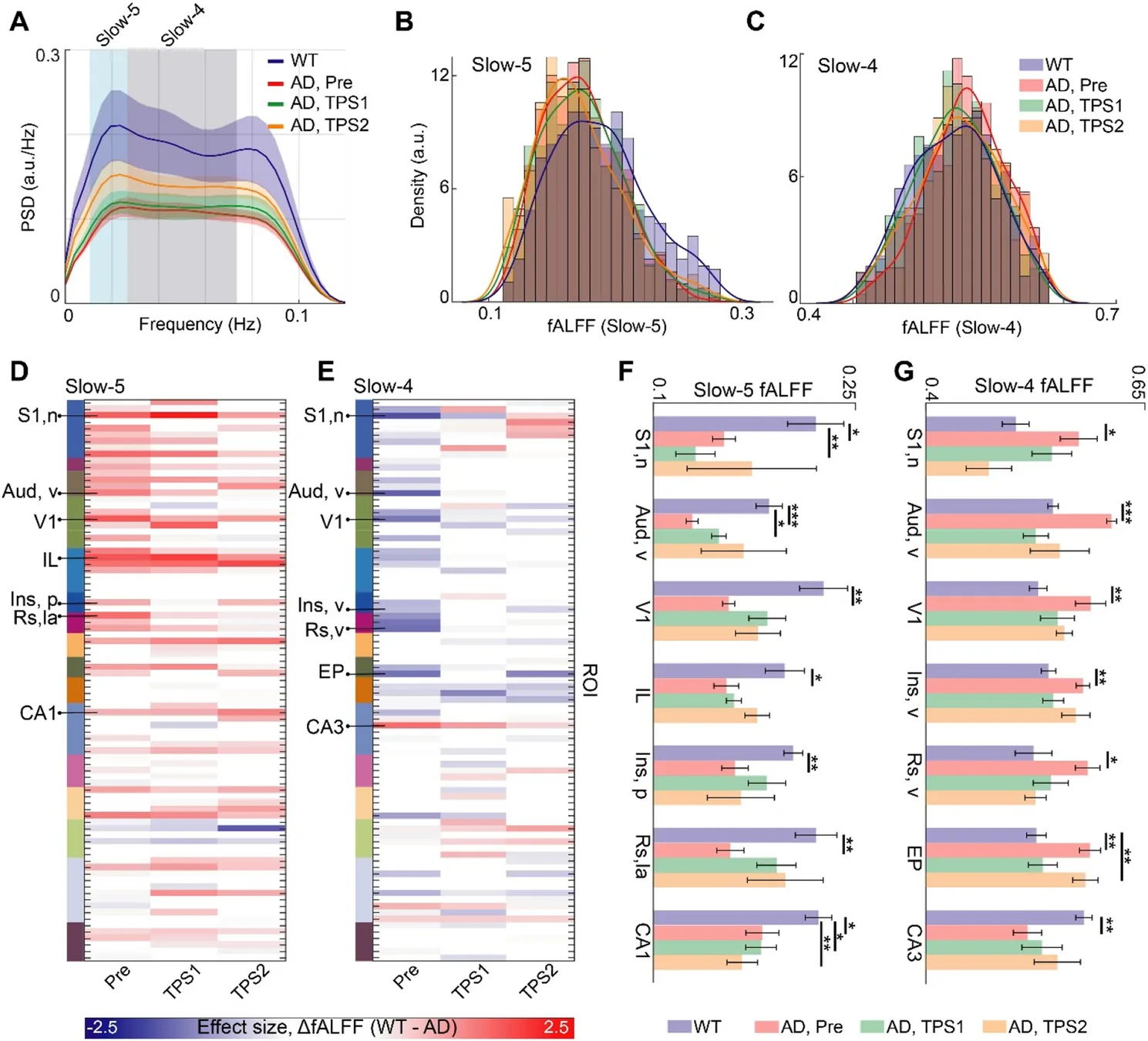
TPS partially normalizes band-specific fractional amplitude of low-frequency fluctuations (fALFF) in 3×Tg-AD mice.** A** Group-averaged power-spectral density (PSD) pooled across all ROIs (WT, *n* = 8; AD, *n* = 6). Shaded regions indicate the slow-5 (0.01–0.027 Hz, blue) and slow-4 (0.027–0.073 Hz, grey) bands. **B-C** Whole-brain fALFF distribution for the slow-5 (B) and slow-4 (C) bands across four conditions: WT, AD before stimulation (AD-Pre), AD after one TPS train (AD-TPS1) and AD after two TPS trains (AD-TPS2). **D-E** Cohen’s *d* effect-size maps comparing WT with AD-Pre, AD-TPS1 and AD-TPS2 for slow-5 (D) and slow-4 (E) fALFF values for each region. ROIs are colorcoded by functional network as in Fig. 1d. **F-G** Mean slow-5 (F) and slow-4 (G) fALFF for the ROIs exhibiting largest baseline differences between WT and 3×Tg-AD. Mean ± s.e.m.; unpaired two-tailed t-test (WT vs. AD-Pre) and paired two-tailed t-tests within the AD cohort (Pre vs. TPS1 vs. TPS2), all FDR-corrected; (*) *p* < 0.05, (**) *p* < 0.01, (***) *p* < 0.001. S1, n, primary somatosensory area, nose; Aud, v, ventral auditory area; V1, primary visual area; IL, infralimbic area; Ins, p, agranular insula, posterior; Rs, la; retrosplenial area, lateral agranular; CA1, hippocampus CA1; Ins, v, agranular insula, ventral; Rs, v, retrosplenial area, ventral; EP, endopiriform nucleus; CA3, hippocampus CA3

The observed PSD alterations motivated a finer-grained, band-resolved examination of spontaneous BOLD fluctuations. We thus quantified the fractional amplitude of low-frequency fluctuations (fALFF) within two canonical frequency bands, slow-5 (0.01–0.027 Hz) and slow-4 (0.027–0.073 Hz), each associated with distinct physiological processes (Fig. 3a-c). Slow-5 primarily captures large-scale circuit and baseline vascular dynamics, whereas slow-4 tracks locally driven neurovascular oscillations prominent in sensory-motor areas [19, 20, 22]. At baseline, 3×Tg-AD mice exhibited significantly reduced slow-5 fALFF across multiple cortical regions including somatosensory, auditory, visual, infralimbic, retrosplenial areas, and insula, whereas slow-4 power was typically increased, particularly in the endopiriform nucleus and reduced selectively in hippocampal CA3 (Fig. 3d-g). Following TPS, the aberrant fractional power distribution seen in 3×Tg-AD mice shifted toward WT levels across both frequency bands, markedly reducing genotype differences across the whole brain (Fig. 3d-g). Interestingly, while the hippocampal CA1 region remained mostly unaffected, other regions demonstrated a clear shift toward normal levels, which was particularly evident after the second TPS application. Even CA3, which showed initial reductions in slow-4 power, exhibited partial normalization.

### Cingulate and caudoputamen network activity partially normalizes following TPS

**Fig. 4:**
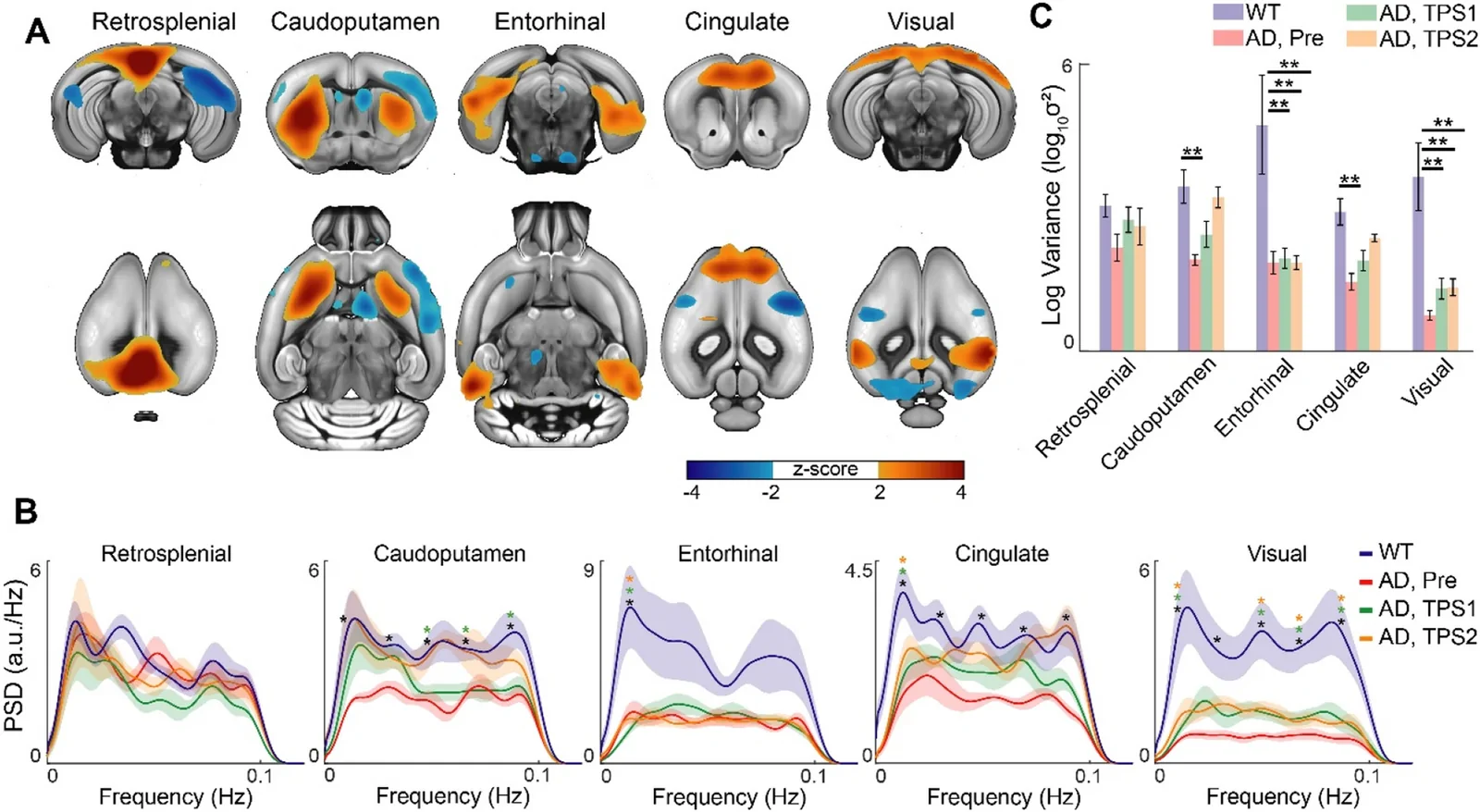
TPS modulates low-frequency BOLD dynamics at network-level in cingulate and caudoputamen networks but not in entorhinal and visual.** A** Spatial maps of independent components overlapping with retrosplenial, caudoputamen, entorhinal, cingulate, and visual networks, identified from concatenated resting-state data (WT, *n* = 8; 3×Tg-AD, *n* = 6). **B** Power-spectral density (PSD) of the component time series across conditions: WT, AD before stimulation (AD-Pre), AD after one TPS train (AD-TPS1) and AD after two trains (AD-TPS2). Shaded envelopes, s.e.m. Frequency bins with significant differences are marked and color-coded (black, WT vs. AD-Pre; green, WT vs. AD-TPS1; yellow, WT vs. AD-TPS2; unpaired t-test for WT vs. AD-Pre and paired two-sided t-test with FDR correction for within-AD comparisons; (*) *p* < 0.05, (**) *p* < 0.01). **C** Variance of the component time courses for the same four conditions. Mean ± s.e.m.; unpaired t-test (WT vs. AD-Pre) and paired two-sided t-test with FDR correction for within-AD comparisons; (*) *p* < 0.05, (**) *p* < 0.01, (***) *p* < 0.001

Building upon the findings of regional spectral power normalization by TPS, we next sought to determine whether similar restorative effects extend to coherent, distributed brain networks. Traditional FC analyses primarily examine specific regions or individual connections, potentially missing broader, network-level dynamics crucial to understanding AD pathology. To address this limitation, we applied independent component analysis (ICA), a data-driven approach that identifies spatially distinct networks by decomposing whole-brain BOLD signals into functionally independent components. Using ICA, we identified 13 biologically meaningful networks (from an initial set of 15 components; SFig. 5). At baseline, differences between 3×Tg-AD and WT mice emerged in the caudoputamen, entorhinal, cingulate, and visual networks (Fig. 4). Specifically, these networks showed reduced variance in their network-level time series and lower overall PSD in 3×Tg-AD mice compared to WT controls. Notably, the retrosplenial network, which is integral to the DMN, did not exhibit significant differences between WT and 3×Tg-AD mice (Fig. 4b, c). Following TPS intervention, AD-associated deficits in caudoputamen and cingulate networks partially normalized towards WT levels, with improvements observed after the first TPS session and further following the second. Conversely, the entorhinal network remained unaffected by TPS, while the visual network demonstrated partial normalization, approaching but not fully reaching WT characteristics after two TPS sessions.

### Non-linear emergence of TPS-induced signal alterations

**Fig. 5:**
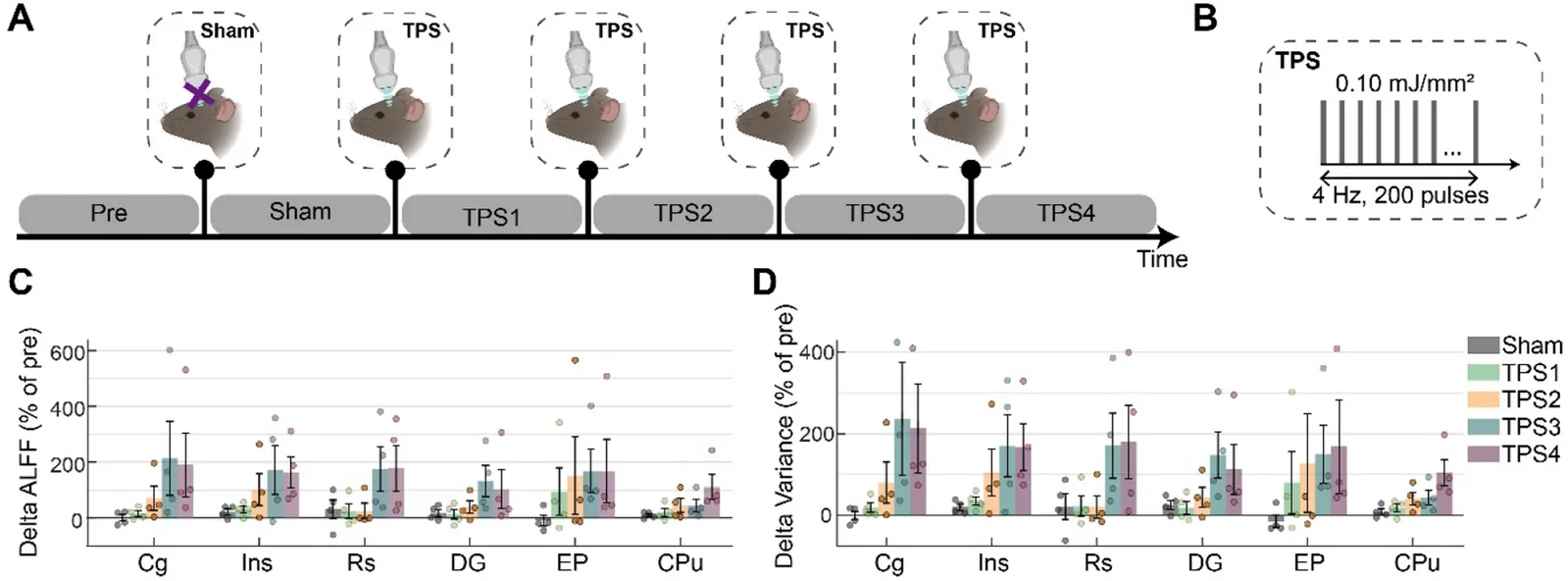
Non-linear emergence of TPS effects in 3×Tg-AD mice.** A–B **Experimental timeline for the fixed-intensity paradigm. Each session began with a baseline resting-state fMRI scan (Pre) followed by sham handling. Mice then received four consecutive medium-intensity TPS trains (0.1 mJ mm^− 2^, 200 pulses), each immediately followed by a 10-min resting-state fMRI acquisition (TPS1–TPS4). **C–D **Changes in regional amplitude of low-frequency fluctuations (ALFF) and BOLD signal variance (mean ± s.e.m.; *n* = 4) relative to baseline, plotted across successive scans for TPS-responsive regions. Cg, anterior cingulate area dorsal part; Ins, agranular insular area dorsal part; Rs, retrosplenial area dorsal part; DG, dentate gyrus; EP, endopiriform nucleus; CPu, caudoputamen

To better understand the temporal emergence of TPS effects across repeated stimulation trains, we employed a complementary fixed-intensity stimulation protocol. In this design, 3×Tg-AD mice (*n* = 4) underwent four consecutive medium-intensity TPS trains (0.1 mJ/mm^− 2^; 200 pulses each), with each train followed by a 10-minute resting-state fMRI acquisition. An initial baseline scan and sham stimulation were also included (Fig. 5a–b). Given the limited sample size, this experiment was intended to provide an exploratory view of the temporal profile of TPS-associated modulation rather than to define an exact physiological threshold. Our analysis revealed that regional spectral amplitude (ALFF) and signal variance remained largely unchanged after the first two TPS trains in several canonical TPS-responsive regions. In contrast, a more pronounced shift emerged after the third stimulation, with signal properties in previously identified TPS-responsive regions, such as the insula, cingulate, and caudoputamen, normalizing toward WT-like levels (Fig. 5c-d). These changes were sustained, but not further increased, during the fourth scan, suggesting a non-linear and region-dependent response profile rather than a simple monotonic increase across successive trains (Fig. 5c-d, SFig. 6). Notably, the endopiriform nucleus showed an earlier response pattern, with detectable changes already after the first stimulation.

### Longitudinal TPS induces sustained rebalancing of low-frequency BOLD spectral dynamics

**Fig. 6:**
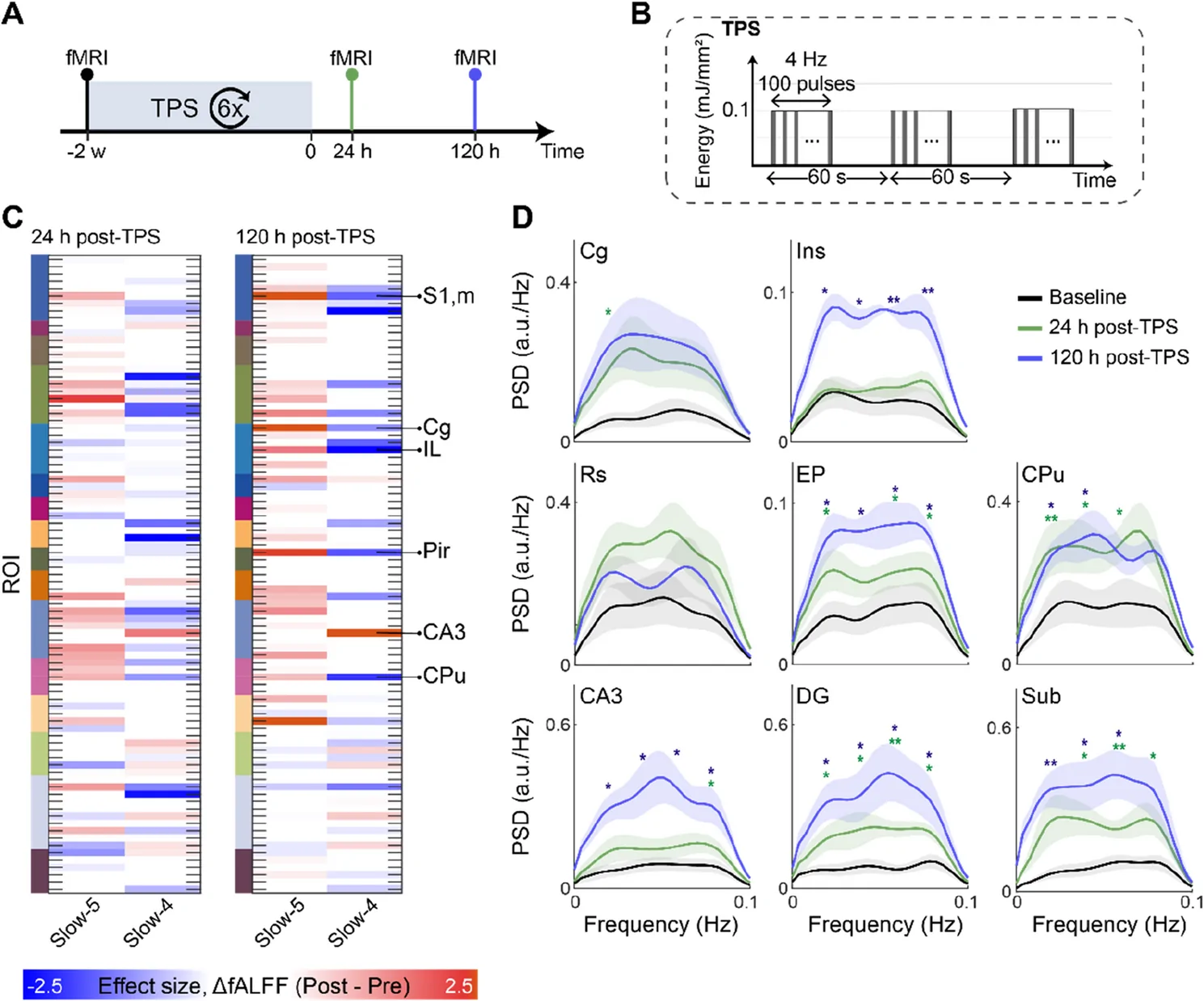
TPS-modulated low-frequency BOLD spectral signatures persist after longitudinal stimulation. **A** Experimental timeline. Mice underwent a baseline rs-fMRI scan followed by six TPS sessions over 2 weeks; rs-fMRI was repeated 24 h and 120 h after the final stimulation. **B** Longitudinal stimulation parameters. Each session comprised medium-intensity TPS trains (0.1 mJ mm^− 2^; 300 pulses; 60 s inter-burst interval). **C** ROI-wise Cohen’s *d* effect-size maps (*n* = 5) for slow-5 and slow-4 fALFF comparing baseline with 24 h post-TPS and 120 h post-TPS. Longitudinal TPS recapitulated the acute spectral shift, with increased slow-5 fALFF and decreased slow-4 fALFF across most ROIs, consistent with a redistribution of power toward slower frequencies. ROIs are color-coded by functional network as in Fig. 1d. **D** ROI power spectral density (PSD) in the low-frequency BOLD band (0.008–0.10 Hz) at baseline, 24 h post-TPS, and 120 h post-TPS (mean ± s.e.m., *n* = 5). Stars indicate frequency bins showing significant differences relative to baseline (green, baseline vs. 24 h; blue, baseline vs. 120 h; paired two-sided *t*-test with FDR correction, (*) *p* < 0.05, (**) *p* < 0.01). Cg, anterior cingulate area dorsal part; Ins, agranular insular area dorsal part; Rs, retrosplenial area dorsal part; EP, endopiriform nucleus; CPu, caudoputamen; CA3, hippocampus CA3; DG, dentate gyrus; Sub, subiculum

To determine whether the spectral rebalancing observed after acute TPS reflects a transient state shift or a more durable change in low-frequency BOLD dynamics, we implemented a longitudinal stimulation paradigm in 3×Tg-AD mice (*n* = 5). Animals underwent a baseline resting-state fMRI scan followed by six TPS sessions delivered over 2 weeks and were re-imaged 24 h and 120 h after the final stimulation (Fig. 6a, b).

Across the brain, longitudinal TPS recapitulated the band-specific redistribution of low-frequency power observed after acute stimulation. ROI-wise effect-size maps revealed a widespread increase in slow-5 fALFF (0.01–0.027 Hz) accompanied by a corresponding reduction in slow-4 fALFF (0.027–0.073 Hz) at 24 h post-TPS, with a broadly similar pattern maintained at 120 h (Fig. 6c). Notably, the hippocampal CA3 subfield again showed an increase in slow-4 fALFF, consistent with the region-specific directionality observed in the acute protocol (Fig. 6c).

These band-resolved changes were mirrored in the full power spectra. ROI PSD analyses demonstrated elevated low-frequency BOLD power at 24 h post-TPS in regions previously identified as TPS-responsive, including cingulate cortex, insula, caudoputamen, and endopiriform nucleus, and these increases remained evident at 120 h (Fig. 6d; SFig. **7**). The temporal trajectory was region-dependent: in some areas (for example, insula and endopiriform nucleus) the spectral power increase further strengthened between 24 h and 120 h, whereas in others (for example, cingulate cortex and caudoputamen) it was largely stable across the two post-treatment time points (Fig. 6d). Importantly, hippocampal regions that showed minimal acute changes exhibited a delayed increase in low-frequency power after longitudinal TPS, detectable at 24 h and more pronounced at 120 h (Fig. 6d; SFig. **8**). Together, these findings indicate that TPS-induced modulation of low-frequency BOLD dynamics persists for at least 5 days after the final session and is accompanied by delayed hippocampal spectral changes under a multi-session regimen.

### Longitudinal TPS is accompanied by preserved exploratory drive and an exploration-adjusted increase in novel object preference in aged 3×Tg-AD mice

**Fig. 7:**
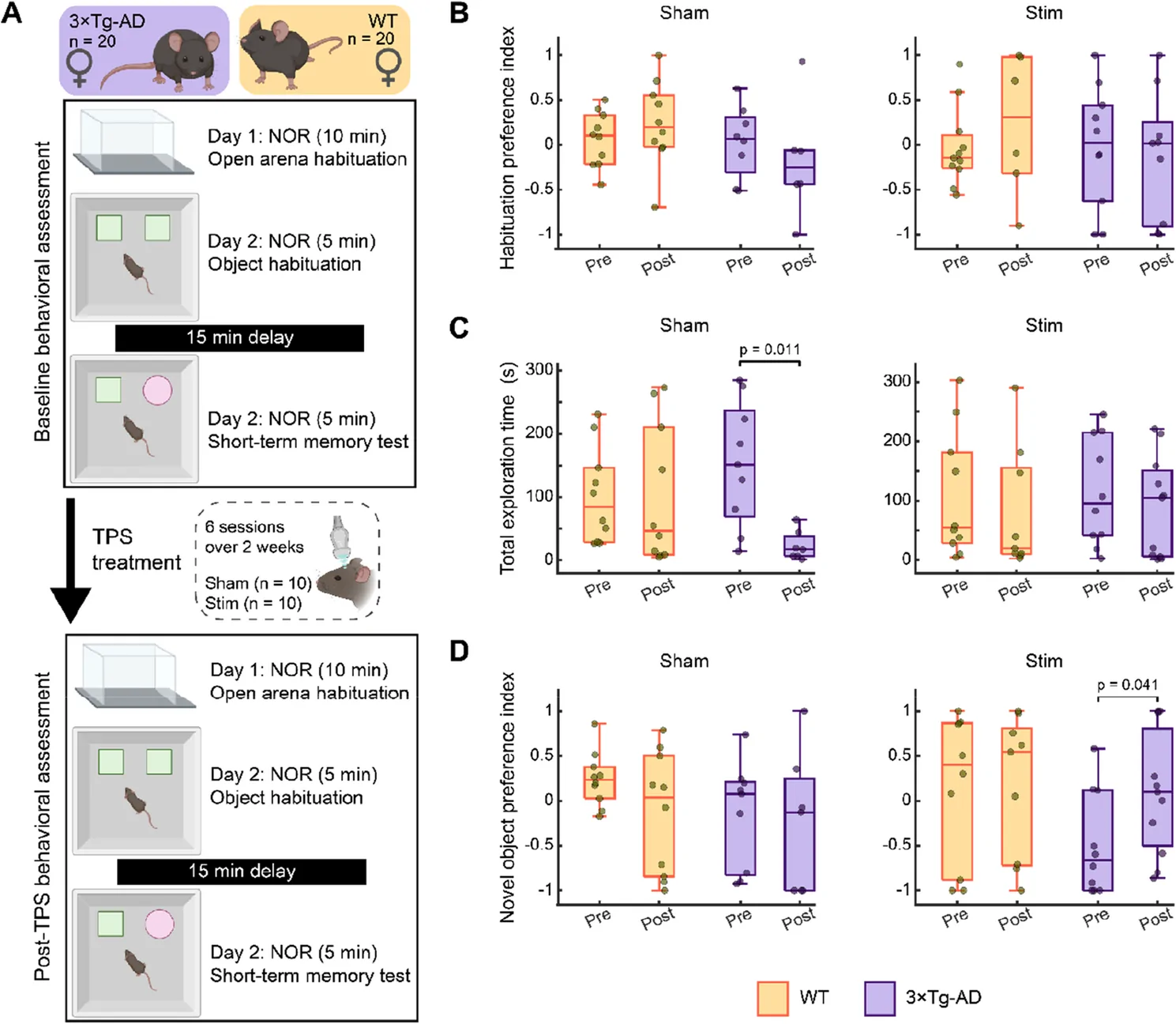
Longitudinal assessment of TPS on exploratory drive and recognition memory.** A** Schematic of the experimental design. Advanced-age female wild-type (WT, *n* = 20) and 3×Tg-AD (*n* = 20) mice underwent a baseline novel object recognition (NOR) assessment. Mice were then randomized to receive either active TPS (Stim, *n* = 10 per genotype) or sham treatment (Sham, *n* = 10 per genotype) across six sessions over two weeks, followed by a post-treatment NOR assessment. **B** Habituation preference index, showing no innate object or side bias during the identical-object phase across cohorts and time points. **C** Total object exploration time during the test phase. 3×Tg-AD mice subjected to the sham procedure showed a post-treatment reduction in exploratory behavior, whereas this decline was not observed in TPS-treated 3×Tg-AD mice. **D** Novel object preference index assessing short-term recognition memory. TPS-treated 3×Tg-AD mice showed a positive post-treatment shift in novel object preference, whereas sham-treated 3×Tg-AD mice did not improve. Preference index was analyzed using genotype-specific linear mixed-effects models, with total exploration time included as a covariate to account for differences in exploratory drive. Planned model-based contrasts were used to assess pre-to-post changes within each treatment group. Data are presented as box plots showing median and interquartile range, with individual data points overlaid. Displayed *p* values indicate the corresponding planned statistical comparisons

To examine whether repeated TPS was accompanied by behavioral changes, we performed a longitudinal novel object recognition (NOR) assessment in a separate cohort of aged female 3×Tg-AD mice (*n* = 20) and age-matched WT controls (*n* = 20) assigned to either TPS or sham treatment under the same 2-week stimulation schedule (Fig. 7a). This design enabled evaluation of treatment-related changes in recognition memory, while also accounting for potential alterations in exploratory drive across genotypes. The task further allowed us to assess possible innate side or object bias during the object habituation phase. Habituation preference indices remained centered around zero across genotypes, treatment groups, and time points (Fig. 7b), indicating that the object sets used for baseline and post-treatment testing were behaviorally neutral and that neither repeated handling nor stimulation introduced a systematic exploration bias.

We next assessed total object exploration time during the test phase to evaluate general changes in exploratory drive, locomotor activity, and motivational state. In WT mice, total exploration remained statistically stable over time in both sham-treated and TPS-treated animals, despite substantial inter-animal variability reflecting the impact of advanced age on individual behavioral trajectories (Fig. 7c). In contrast, sham-treated 3×Tg-AD mice showed a significant reduction in total object exploration at the post-treatment time point, decreasing from 152.7 ± 98.3 s at baseline to 22.8 ± 23.1 s after sham treatment (paired one-sided t-test, *p* = 0.011). TPS-treated 3×Tg-AD mice did not exhibit such a decline, with total exploration changing from 114.3 ± 90.6 s at baseline to 88.4 ± 85.6 s after treatment (paired one-sided t-test, *p* = 0.596). Thus, the loss of object-directed exploratory engagement observed in sham-treated 3×Tg-AD mice was not evident in TPS-treated 3×Tg-AD mice, suggesting that repeated TPS helped maintain exploratory drive in the aged transgenic cohort.

We then evaluated the primary cognitive outcome: the ability to discriminate a novel object from a familiar one across the longitudinal study (Fig. 7d). Because preference index values can be influenced by the total amount of object-directed exploration, novel object preference was analyzed using genotype-specific linear mixed-effects models. For each genotype, the model included treatment, time, and their interaction as fixed effects, total exploration time as a continuous covariate, and animal identity as a random intercept. Planned model-based contrasts were then used to test post-treatment versus pre-treatment changes within each treatment group, while controlling for total exploration time.

In WT mice, novel object preference remained statistically stable from baseline to post-treatment in both treatment groups after controlling for total exploration time. Neither sham-treated WT mice nor TPS-treated WT mice showed a significant pre-to-post change in preference index (linear mixed-effects model contrast: Sham, β = −0.373, 95% CI: −0.892 to 0.146, *p* = 0.153; TPS, β = −0.008, 95% CI: −0.555 to 0.539, *p* = 0.977). The treatment-by-time interaction was also not significant in WT mice (linear mixed-effects model interaction contrast, β = 0.365, 95% CI: −0.389 to 1.120, *p* = 0.332), indicating that TPS did not alter NOR performance in healthy aged controls.

In 3×Tg-AD mice, sham-treated animals did not show a significant pre-to-post improvement in novel object preference after controlling for total exploration time (linear mixed-effects model contrast, β = −0.242, 95% CI: −0.940 to 0.456, *p* = 0.485). By contrast, TPS-treated 3×Tg-AD mice showed a significant increase in novel object preference in the exploration-adjusted model (linear mixed-effects model contrast, β = 0.552, 95% CI: 0.023 to 1.082, *p* = 0.0415). The direct treatment-by-time contrast within 3×Tg-AD mice showed a trend-level difference between TPS and sham trajectories (linear mixed-effects model interaction contrast, β = 0.795, 95% CI: −0.051 to 1.641, *p* = 0.0647), suggesting that the improvement in novel object discrimination was preferentially expressed in the stimulated transgenic cohort.

Together, these behavioral findings indicate that aged sham-treated 3×Tg-AD mice showed a decline in exploratory engagement over time, whereas TPS-treated 3×Tg-AD mice did not exhibit this loss of object-directed exploration. Importantly, the increase in novel object preference in TPS-treated 3×Tg-AD mice remained evident in a linear mixed-effects model controlling for total exploration time, supporting the interpretation that the behavioral improvement was not simply explained by altered exploratory drive. While the variability inherent to aged and pathologically burdened cohorts [45] warrants cautious interpretation, these findings indicate that TPS-associated network modulation was accompanied by preserved object-directed exploration and an exploration-adjusted increase in novel object preference. Given the trend-level treatment-by-time interaction and the use of separate imaging and behavioral cohorts, these behavioral data should be interpreted as preliminary supportive evidence rather than proof of restored recognition memory.

## Discussion

The presented results demonstrate that TPS can acutely and longitudinally modulate large-scale network-level abnormalities in the 3×Tg-AD mouse model of AD. Untreated 3×Tg-AD mice exhibited characteristic AD-like abnormalities, namely widespread functional disconnections spanning default-mode, insular, and striatal circuits, together with a marked reduction in low-frequency BOLD oscillatory power and a redistribution of spectral energy from the slow-5 (0.01–0.027 Hz) to the slow-4 (0.027–0.073 Hz) band. These disruptions mirror observations in prodromal AD patients, for which selective loss of slow-5 amplitude in the posterior cingulate and precuneus forecasts memory decline and tracks Aβ burden [46]. By delivering brief trains of acoustic impulses, TPS shifted the spectral composition of these circuits toward WT-like profiles, with the strongest rebounds in the cingulate, insula, caudoputamen, and endopiriform nucleus, whereas the heavily burdened hippocampus remained less affected in the acute setting. Critically, the fixed-intensity paradigm suggested that these improvements followed a non-linear and region-dependent temporal profile rather than a simple monotonic increase across successive stimulation trains. While some regions showed earlier responsiveness, the clearest and most consistent effects across canonical TPS-responsive regions emerged only after repeated stimulation. Given the limited sample size and design of this experiment, however, we cannot distinguish whether this pattern reflects repeated stimulation per se, cumulative delivered acoustic energy, or another region-specific integration process.

Importantly, the longitudinal stimulation paradigm showed that these effects are not confined to the immediate post-stimulation window. The principal effects observed acutely were reproduced under the longitudinal regimen, arguing against a purely transient perturbation and instead supporting a reproducible shift in the intrinsic spectral organization of AD-affected brain networks, at the level of BOLD-derived network metrics. Notably, these changes remained evident 24 h and 120 h after the final stimulation session, with several regions showing equal or stronger normalization at 120 h than at 24 h. Although the present data do not directly demonstrate synaptic plasticity, the persistence and delayed strengthening of the BOLD-derived response suggest that TPS-associated network modulation may outlast the immediate post-stimulation period. This temporal profile is compatible with longer-timescale adaptive processes, but the underlying cellular, synaptic, vascular, or neurovascular mechanisms remain to be tested directly. Importantly, the same principal spectral changes observed acutely were reproduced in separate post-treatment imaging sessions acquired 24 h and 120 h after the final TPS session under the same baseline anesthesia conditions, making it unlikely that the observed acute effects simply reflect an immediate TPS-induced shift in anesthetic depth or arousal state.

The behavioral findings provide complementary functional support for the longitudinal spectral normalization observed via fMRI, particularly within the anterior cingulate cortex and caudoputamen. While novel object recognition is fundamentally supported by medial temporal lobe circuitry, its successful expression in aged and AD-like states also depends on the functional integrity of distributed networks that govern attention, goal-directed investigation, and object-directed exploration. This is most evident in the exploratory behavior: whereas sham-treated 3×Tg-AD mice exhibited a marked reduction in total object sampling over time, TPS-treated 3×Tg-AD mice did not show this decline. This stabilization aligns with the observed normalization of caudoputamen and cingulate network dynamics, suggesting that TPS may help preserve behavioral prerequisites such as attention, motor planning, and novelty-seeking that are necessary for a reliable readout of recognition memory.

Importantly, the positive shift in novel object preference in TPS-treated 3×Tg-AD mice remained significant after controlling for total exploration time in the linear mixed-effects model. This supports the interpretation that the observed change was not simply explained by preserved exploratory drive. At the same time, the direct treatment-by-time contrast between TPS- and sham-treated 3×Tg-AD mice remained trend-level, indicating that the behavioral effect should be interpreted cautiously. Thus, the behavioral data are best viewed as preliminary evidence that TPS-associated network modulation was accompanied by preserved task engagement and an exploration-adjusted increase in novel object preference, rather than as evidence of robust recovery of recognition memory. This pattern is consistent with the staged recovery profile revealed by our longitudinal imaging: executive and motor-related networks may normalize earlier and support active environmental engagement, whereas memory-relevant hippocampal circuitry showed a more delayed rebalancing trajectory. This temporal mismatch suggests that, at the time of behavioral assessment, the machinery supporting exploration and task engagement may have recovered before full stabilization of the underlying memory-encoding signatures.

At the same time, the decline in total exploration observed in the sham-treated 3×Tg-AD group introduces an important interpretational caveat. Because the preference index is a ratio, severely reduced object sampling can inflate variance and amplify the impact of brief or potentially stochastic interactions. Thus, the poor post-treatment discrimination in the sham cohort may reflect both persistent mnemonic dysfunction and incomplete object sampling. By contrast, the preservation of object-directed exploration in TPS-treated 3×Tg-AD mice provides a more stable behavioral basis for interpreting the preference index. The fact that the TPS-associated increase in novel object preference remained significant after including total exploration time as a covariate further supports the interpretation that the observed improvement was not merely driven by differences in exploratory drive.

A notable finding is the differential temporal responsiveness of hippocampal regions. Whereas hippocampal areas showed minimal or absent spectral changes following acute TPS, they became responsive under the longitudinal regimen, with clearer normalization at 120 h than at 24 h after the final stimulation. Several non-mutually exclusive explanations may account for this pattern. First, the hippocampus may harbor a greater pathological burden in the 3×Tg-AD model, such that acute TPS is insufficient to produce detectable BOLD spectral modulation, whereas repeated stimulation may be required for measurable effects [12, 13]. Second, delayed hippocampal changes may arise indirectly through prior modulation of interconnected cortical and subcortical hubs, including cingulate, insular, or striatal regions. Third, hippocampal responses may depend more strongly on slower neurovascular, glial, or synaptic processes that do not peak in the immediate post-stimulation window, consistent with prior ultrasound-based work showing restoration of dentate gyrus long-term potentiation and memory in aged mice [47].

In addition, the delayed hippocampal changes observed here are compatible with the broader idea that repeated ultrasound-based neuromodulation can induce delayed post-stimulation network adaptation [31, 48–50]. The fact that hippocampal spectral normalization was more evident at 120 h than at 24 h is therefore particularly interesting in light of the exploration-adjusted increase in novel object preference observed in TPS-treated 3×Tg-AD mice. Although imaging and behavior were acquired in separate cohorts, the convergence of delayed hippocampal spectral normalization, preserved object-directed exploration, and improved exploration-adjusted novel object preference supports further investigation of whether TPS-associated modulation of memory-relevant circuitry contributes to behavioral outcomes beyond the immediate post-stimulation period.

The findings reinforce the emerging view that AD is driven as much by network dysrhythmia as by proteinopathy [38, 43, 48, 49]. The selective dampening of slow-frequency oscillations observed here is notable because slow rhythms are believed to modulate long-range communication, neurovascular coupling, and clearance pathways such as glymphatic flow [51–53]. Their attenuation in 3×Tg-AD mice likely reflects a confluence of synaptic hypo-excitability, tau-induced axonal transport failure, and microvascular dysfunction, each of which has been implicated in AD [54–56]. The regional specificity of TPS-induced spectral recovery may offer clues to the mechanisms through which TPS acts. Specifically, default-mode hubs (anterior cingulate/retrosplenial cortex) and anterior insula (crucial for default-mode and salience network switching) responded robustly, as did the caudoputamen, a striatal region increasingly recognized as an early tau target. Such responsiveness hints that TPS may be particularly effective at nodes with preserved structural integrity but suppressed excitability. The accompanying preservation of exploratory engagement in the behavioral cohort further raises the possibility that modulation of these distributed circuits may extend beyond mnemonic processing alone and support broader behavioral functions, such as attention, motor planning, and novelty-seeking, that are often degraded in advanced disease states. From a translational standpoint, the non-linear and region-dependent emergence of TPS-mediated changes underscores the need for dose-optimization studies, as sub-threshold regimens may leave clinically relevant circuits unmodulated.

A key question is how TPS can re-establish oscillatory power and network coherence. The biological mechanisms underlying these effects remain unresolved in the present study, and the possibilities discussed below should therefore be regarded as hypotheses rather than direct inferences from our data. One plausible mechanism involves direct modulation of neuronal activity via mechanosensitive ion channels, which are known to respond to rapid pressure changes [57]. While TPS differs from traditional low-intensity focused ultrasound in its use of ultrashort acoustic pulses, similar mechanosensitive pathways may be engaged. Indeed, ultrasound has been shown to stimulate neuronal firing and entrain neural activity across a range of frequencies through such channels [58, 59]. By imposing acoustic pulses with defined intensities, durations, and repetition frequencies, TPS could reset aberrant phase relationships and permit re-emergence of slow network oscillations. A complementary hypothesis involves neurovascular effects. Low-intensity ultrasound has been shown to transiently increase microvascular perfusion and fine-tune neurovascular coupling, thereby amplifying the hemodynamic responses of underlying neural activity [60, 61]. TPS has also been shown to induce hemodynamic changes in the murine brain [37]. Repeated stimulation may therefore progressively reshape the vascular and metabolic support of vulnerable circuits, enabling the corresponding low-frequency BOLD signature to remain normalized beyond the acute post-stimulation period. Finally, glial modulation remains another possibility, particularly given prior ultrasound-based studies showing microglial recruitment and Aβ clearance [62]. However, the present study did not assess glial activation, synaptic plasticity, vascular reactivity, or molecular pathology; these mechanisms therefore remain speculative and require direct experimental validation.

The findings of this study concur with emerging human data. Pilot TPS trials in mild to moderate AD have reported cognitive gains accompanied by heightened parietal–hippocampal connectivity and sustained cortical glucose uptake, while non-invasive gamma-frequency light and sound entrainment likewise increase default-mode coherence and slow ventricular expansion [63–65]. Together, these convergent findings suggest that re-synchronizing large-scale networks, whether through shockwaves, ultrasound, electromagnetic pulses, or sensory entrainment, can translate into measurable functional benefits even in the face of ongoing pathology.

These conclusions come with several caveats. First, although the spectral changes persisted for at least 120 h after the final stimulation and behavioral testing indicated an exploration-adjusted improvement in novel object preference in TPS-treated 3×Tg-AD mice, the behavioral assessment was restricted to an immediate short-term NOR readout and could not be extended to remote memory. Second, behavioral testing was performed in a separate cohort, precluding direct subject-level correspondence between network rescue and cognitive outcome. Third, the reduction in object sampling in sham-treated 3×Tg-AD mice complicates interpretation of the ratio-based preference index, because severe declines in exploration can inflate variance and reduce the stability of discrimination measures. We addressed this by including total exploration time as a covariate in the mixed-effects analysis; nevertheless, future studies should include additional behavioral tasks and longer-term follow-up to confirm whether TPS produces durable improvements in recognition memory. Fourth, the biological mechanisms underlying TPS-induced network modulation were not directly examined in the present study; thus, molecular and cellular correlates of synaptic plasticity, neurovascular regulation, and glial responses remain to be established. Fifth, sample sizes were modest, and faster neuronal rhythms that may interact with the restored low-frequency dynamics were not examined. Importantly, the 3×Tg-AD mouse model exhibits marked sexual dimorphism, with female mice generally accumulating higher Aβ and tau burdens [66]. Although TPS-induced rescue of brain circuitry was observed in both sexes, the imaging cohort remained too small to detect subtler sex-specific differences, and the behavioral study was limited to aged female mice. Future studies should therefore combine longitudinal imaging with broader and longer-term behavioral follow-up, electrophysiological and molecular readouts, and testing across additional AD models, disease stages, and sexes to define the boundary conditions of TPS responsiveness.

Nonetheless, by demonstrating that a non-invasive single-pulse shockwave protocol can recalibrate the spectral signature and network coherence of a brain rendered dysfunctional by Aβ and tau, this study supports TPS as a potentially tractable circuit-level neuromodulatory strategy for AD. The convergence of acute and longitudinal imaging findings, together with preserved object-directed exploration and an exploration-adjusted increase in novel object preference in a separate behavioral cohort, suggests that TPS-associated network modulation may be accompanied by preliminary behavioral support. As the field gradually shifts from an exclusive focus on Aβ reduction toward multifaceted interventions, TPS and related neuromodulatory strategies provide a framework for testing whether modulation of vulnerable circuit dynamics can influence disease-relevant functional outcomes. Whether these network-level effects translate into durable cognitive benefit will require larger, well-controlled preclinical and clinical studies with longitudinal behavioral, molecular, electrophysiological, and neurovascular readouts.

## Conclusions

In summary, the selective loss of low-frequency oscillatory power and network coherence contributes to impaired large-scale communication in AD. We propose that TPS partially rebalances these BOLD-derived dynamics in a non-linear, region-dependent manner, highlighting neuromodulation of oscillatory brain activity as a candidate approach for modulating AD-related network dysfunction. By linking frequency-resolved fMRI metrics with longitudinal stimulation and behavioral readouts, this work provides a framework for evaluating how circuit-level interventions influence vulnerable brain networks over time. Further studies combining longer-term behavioral follow-up with molecular, electrophysiological, and neurovascular readouts will be needed to define the mechanisms, durability, and therapeutic boundaries of TPS-induced network modulation. 

## Electronic Supplementary Material

Below is the link to the electronic supplementary material.

Supplementary Material 1.

## Data Availability

The data and code that support the findings of this study are available from the corresponding author upon request. All data supporting the findings of this study are found within the paper and its Supplementary Information.

## Abbreviations

3×Tg-AD: Triple-transgenic Alzheimer’s disease mouse model
Aβ: Amyloid-β
AD: Alzheimer’s disease
ALFF: Amplitude of low-frequency fluctuations
APP: Amyloid precursor protein (Swedish mutation)
BOLD: Blood-oxygen-level-dependent (signal)
CA1/CA3: Cornu Ammonis fields 1/3 of hippocampus
CCF: Common Coordinate Framework (Allen Mouse Brain)
Cg: Anterior cingulate area (dorsal part, unless noted)
CPu: Caudoputamen
DG: Dentate gyrus
DMN: Default mode network
EEG: Electroencephalography
EP: Endopiriform nucleus
EPI: Echo-planar imaging
fALFF: Fractional amplitude of low-frequency fluctuations
FC: Functional connectivity
FDR: False discovery rate
fMRI: Functional magnetic resonance imaging
FWHM: Full width at half maximum
GE-EPI: Gradient-echo echo-planar imaging
HT7: Human tau monoclonal antibody clone HT7
Hypo: Lateral hypothalamic area
ICA: Independent component analysis
IC: Inferior colliculus
IHC: Interhemispheric connectivity
IL: Infralimbic area
Ins: Agranular insular area (posterior/dorsal/ventral as specified)
NA: Numerical aperture
OCT: Optimal cutting temperature (compound)
PBS: Phosphate-buffered saline
PBST: PBS with Triton X-100
PFA: Paraformaldehyde
PSD: Power spectral density
RARE: Rapid acquisition with relaxation enhancement
ROI: Region of interest
Rs: Retrosplenial cortex (lateral agranular/dorsal/ventral as specified)
s.e.m.: Standard error of the mean
tACS: Transcranial alternating-current stimulation
TE: Echo time
TMS: Transcranial magnetic stimulation
TPS: Transcranial pulse stimulation
TR: Repetition time
V1: Primary visual area
WT: Wild-type

## References

[1] Al-Ezzi A, Arechavala RJ, Butler R, Nolty A, Kang JJ, Shimojo S, et al. Disrupted brain functional connectivity as early signature in cognitively healthy individuals with pathological CSF amyloid/tau. Commun Biology. 2024;7(1):1037. 10.1038/s42003-024-06673-w PMC11344156 39179782

[2] Selkoe DJ. Early network dysfunction in Alzheimer’s disease. Science. 2019;365(6453):540–1. 31395769 10.1126/science.aay5188

[3] Jullienne A, Trinh MV, Obenaus A. Neuroimaging of Mouse Models of Alzheimer’s Disease. Biomedicines. 2022;10(2):305. 35203515 10.3390/biomedicines10020305 PMC8869427

[4] Eyler LT, Elman JA, Hatton SN, Gough S, Mischel AK, Hagler DJ, et al. Resting state abnormalities of the default mode network in mild cognitive impairment: a systematic review and meta-analysis. J Alzheimer’s Disease. 2019;70(1):107–20. 31177210 10.3233/JAD-180847 PMC6697380

[5] Chiesa PA, Cavedo E, Vergallo A, Lista S, Potier M-C, Habert M-O, et al. Differential default mode network trajectories in asymptomatic individuals at risk for Alzheimer’s disease. Alzheimer’s Dement. 2019;15(7):940–50. 31113760 10.1016/j.jalz.2019.03.006

[6] Palmqvist S, Schöll M, Strandberg O, Mattsson N, Stomrud E, Zetterberg H, et al. Earliest accumulation of β-amyloid occurs within the default-mode network and concurrently affects brain connectivity. Nat Commun. 2017;8(1):1214. 29089479 10.1038/s41467-017-01150-x PMC5663717

[7] Beason-Held LL, Goh JO, An Y, Kraut MA, O’Brien RJ, Ferrucci L, et al. Changes in brain function occur years before the onset of cognitive impairment. J Neurosci. 2013;33(46):18008–14. 24227712 10.1523/JNEUROSCI.1402-13.2013 PMC3828456

[8] Crimins JL, Pooler A, Polydoro M, Luebke JI, Spires-Jones TL. The intersection of amyloid β and tau in glutamatergic synaptic dysfunction and collapse in Alzheimer’s disease. Ageing Res Rev. 2013;12(3):757–63. 23528367 10.1016/j.arr.2013.03.002 PMC3735866

[9] Jack CR Jr., Knopman DS, Jagust WJ, Shaw LM, Aisen PS, Weiner MW, et al. Hypothetical model of dynamic biomarkers of the Alzheimer’s pathological cascade. Lancet Neurol. 2010;9(1):119–28. 20083042 10.1016/S1474-4422(09)70299-6 PMC2819840

[10] Bloom GS. Amyloid-β and tau: the trigger and bullet in Alzheimer disease pathogenesis. JAMA Neurol. 2014;71(4):505–8. 24493463 10.1001/jamaneurol.2013.5847 PMC12908160

[11] Mandino F, Yeow LY, Bi R, Sejin L, Bae HG, Baek SH, et al. The lateral entorhinal cortex is a hub for local and global dysfunction in early Alzheimer’s disease states. J Cereb Blood Flow Metab. 2022;42(9):1616–31. 35466772 10.1177/0271678X221082016 PMC9441719

[12] Oddo S, Caccamo A, Shepherd JD, Murphy MP, Golde TE, Kayed R, et al. Triple-transgenic model of Alzheimer’s disease with plaques and tangles: intracellular Abeta and synaptic dysfunction. Neuron. 2003;39(3):409–21. 12895417 10.1016/s0896-6273(03)00434-3

[13] Billings LM, Oddo S, Green KN, McGaugh JL, LaFerla FM. Intraneuronal Abeta causes the onset of early Alzheimer’s disease-related cognitive deficits in transgenic mice. Neuron. 2005;45(5):675–88. 15748844 10.1016/j.neuron.2005.01.040

[14] Mantini D, Perrucci MG, Del Gratta C, Romani GL, Corbetta M. Electrophysiological signatures of resting state networks in the human brain. Proc Natl Acad Sci U S A. 2007;104(32):13170–5. 17670949 10.1073/pnas.0700668104 PMC1941820

[15] Zuo XN, Di Martino A, Kelly C, Shehzad ZE, Gee DG, Klein DF, et al. The oscillating brain: complex and reliable. NeuroImage. 2010;49(2):1432–45. 19782143 10.1016/j.neuroimage.2009.09.037 PMC2856476

[16] Courtney SM, Hinault T. When the time is right: Temporal dynamics of brain activity in healthy aging and dementia. Prog Neurobiol. 2021;203:102076. 34015374 10.1016/j.pneurobio.2021.102076

[17] Wiesman AI, Murman DL, Losh RA, Schantell M, Christopher-Hayes NJ, Johnson HJ, et al. Spatially resolved neural slowing predicts impairment and amyloid burden in Alzheimer’s disease. Brain. 2022;145(6):2177–89. 35088842 10.1093/brain/awab430 PMC9246709

[18] Hrybouski S, Das SR, Xie L, Brown CA, Flamporis M, Lane J, et al. BOLD amplitude correlates of preclinical Alzheimer’s disease. Neurobiol Aging. 2025;150:157–71. 40138942 10.1016/j.neurobiolaging.2025.03.007

[19] Yang L, Yan Y, Li Y, Hu X, Lu J, Chan P, et al. Frequency-dependent changes in fractional amplitude of low-frequency oscillations in Alzheimer’s disease: a resting-state fMRI study. Brain Imaging Behav. 2020;14(6):2187–201. 31478145 10.1007/s11682-019-00169-6

[20] Li Y, Yao H, Lin P, Zheng L, Li C, Zhou B, et al. Frequency-Dependent Altered Functional Connections of Default Mode Network in Alzheimer’s Disease. Front Aging Neurosci. 2017;9:259. 28824420 10.3389/fnagi.2017.00259 PMC5540901

[21] Zeng Q, Luo X, Li K, Wang S, Zhang R, Hong H, et al. Distinct Spontaneous Brain Activity Patterns in Different Biologically-Defined Alzheimer’s Disease Cognitive Stage: A Preliminary Study. Front Aging Neurosci. 2019;11:350. 32009939 10.3389/fnagi.2019.00350 PMC6980867

[22] Han Y, Wang J, Zhao Z, Min B, Lu J, Li K, et al. Frequency-dependent changes in the amplitude of low-frequency fluctuations in amnestic mild cognitive impairment: a resting-state fMRI study. NeuroImage. 2011;55(1):287–95. 21118724 10.1016/j.neuroimage.2010.11.059

[23] Xuena L, Siqi W, Xinqing Z, Zhiqun W, Xiaojie T, Yong H. Abnormal Amplitude of Low-Frequency Fluctuations of Intrinsic Brain Activity in Alzheimer’s Disease. J Alzheimer’s Disease. 2014;40(2):387–97. 24473186 10.3233/JAD-131322

[24] Shah D, Jonckers E, Praet J, Vanhoutte G, Delgado YPR, Bigot C, et al. Resting state FMRI reveals diminished functional connectivity in a mouse model of amyloidosis. PLoS ONE. 2013;8(12):e84241. 24358348 10.1371/journal.pone.0084241 PMC3866274

[25] Manno FAM, Isla AG, Manno SHC, Ahmed I, Cheng SH, Barrios FA, et al. Early Stage Alterations in White Matter and Decreased Functional Interhemispheric Hippocampal Connectivity in the 3xTg Mouse Model of Alzheimer’s Disease. Front Aging Neurosci. 2019;11:39. 30967770 10.3389/fnagi.2019.00039 PMC6440287

[26] Koch G, Altomare D, Benussi A, Bréchet L, Casula EP, Dodich A, et al. The emerging field of non-invasive brain stimulation in Alzheimer’s disease. Brain. 2024;147(12):4003–16. 39562009 10.1093/brain/awae292 PMC11734340

[27] Martorell AJ, Paulson AL, Suk H-J, Abdurrob F, Drummond GT, Guan W, et al. Multi-sensory gamma stimulation ameliorates Alzheimer’s-associated pathology and improves cognition. Cell. 2019;177(2):256–71. e22. 30879788 10.1016/j.cell.2019.02.014 PMC6774262

[28] Beisteiner R, Matt E, Fan C, Baldysiak H, Schönfeld M, Philippi Novak T, et al. Transcranial Pulse Stimulation with Ultrasound in Alzheimer’s Disease-A New Navigated Focal Brain Therapy. Adv Sci (Weinh). 2020;7(3):1902583. 32042569 10.1002/advs.201902583 PMC7001626

[29] Karakatsani ME, Gezginer I, Nozdriukhin D, et al. Transcranial pulse stimulation modulates neuronal activity and functional network dynamics. Brain Stimul. 2025;18(6):1834-1842. 41033413 10.1016/j.brs.2025.09.021 PMC13352088

[30] Matt E, Dörl G, Beisteiner R. Transcranial pulse stimulation (TPS) improves depression in AD patients on state-of-the-art treatment. Alzheimers Dement (N Y). 2022;8(1):e12245. 35169611 10.1002/trc2.12245 PMC8829892

[31] Matt E, Kaindl L, Tenk S, Egger A, Kolarova T, Karahasanović N, et al. First evidence of long-term effects of transcranial pulse stimulation (TPS) on the human brain. J Transl Med. 2022;20(1):26. 35033118 10.1186/s12967-021-03222-5 PMC8760674

[32] Wang QX, Ding SL, Li Y, Royall J, Feng D, Lesnar P, et al. The Allen Mouse Brain Common Coordinate Framework: A 3D Reference Atlas. Cell. 2020;181(4):936–. 32386544 10.1016/j.cell.2020.04.007 PMC8152789

[33] GezginerI Chen Y, Zerbi V, Chen Z, Razansky D. Hypercapnia dissociates neuronal and hemodynamic responses impairing neurovascular coupling and functional brain connectivity. Nat Commun. Published online April 13, 2026. 10.1038/s41467-026-71742-z. 10.1038/s41467-026-71742-z PMC13249851 41974737

[34] Gezginer I, Chen Z, Yoshihara HAI, Dean-Ben XL, Zerbi V, Razansky D. Concurrent optoacoustic tomography and magnetic resonance imaging of resting-state functional connectivity in the mouse brain. Nat Commun. 2024;15(1):10791. 39737925 10.1038/s41467-024-54947-y PMC11685406

[35] Gezginer I, Mazzini G, Le Foll C, Kindler D, Lutz TA, Razansky D. Pancreatic amylin dynamically reconfigures distributed brain networks governing appetite regulation in mice. Mol Metab. 2026;103:102313. 41443331 10.1016/j.molmet.2025.102313 PMC12808607

[36] Radjenovic S, Dörl G, Gaal M, Beisteiner R. Safety of Clinical Ultrasound Neuromodulation. Brain Sci. 2022;12(10):1277. 36291211 10.3390/brainsci12101277 PMC9599299

[37] Karakatsani ME, Nozdriukhin D, Tiemann S, Yoshihara HA, Storz R, Belau M et al. Multimodal imaging of murine cerebrovascular dynamics induced by transcranial pulse stimulation. Alzheimer’s Dement. 2025;21(2):e14511. 39807706 10.1002/alz.14511 PMC11848200

[38] Sheline YI, Raichle ME. Resting state functional connectivity in preclinical Alzheimer’s disease. Biol Psychiatry. 2013;74(5):340–7. 23290495 10.1016/j.biopsych.2012.11.028 PMC3537262

[39] Sheline YI, Raichle ME, Snyder AZ, Morris JC, Head D, Wang S, et al. Amyloid plaques disrupt resting state default mode network connectivity in cognitively normal elderly. Biol Psychiatry. 2010;67(6):584–7. 19833321 10.1016/j.biopsych.2009.08.024 PMC2829379

[40] Zhou J, Greicius MD, Gennatas ED, Growdon ME, Jang JY, Rabinovici GD, et al. Divergent network connectivity changes in behavioural variant frontotemporal dementia and Alzheimer’s disease. Brain. 2010;133(Pt 5):1352–67. 20410145 10.1093/brain/awq075 PMC2912696

[41] Brier MR, Thomas JB, Snyder AZ, Benzinger TL, Zhang D, Raichle ME, et al. Loss of intranetwork and internetwork resting state functional connections with Alzheimer’s disease progression. J Neurosci. 2012;32(26):8890–9. 22745490 10.1523/JNEUROSCI.5698-11.2012 PMC3458508

[42] Lu J, Testa N, Jordan R, Elyan R, Kanekar S, Wang J et al. Functional Connectivity between the Resting-State Olfactory Network and the Hippocampus in Alzheimer’s Disease. Brain Sci. 2019;9(12):338. 31775369 10.3390/brainsci9120338 PMC6955985

[43] Roh JH, Park MH, Ko D, Park KW, Lee DH, Han C, et al. Region and frequency specific changes of spectral power in Alzheimer’s disease and mild cognitive impairment. Clin Neurophysiol. 2011;122(11):2169–76. 21715226 10.1016/j.clinph.2011.03.023

[44] Wang R, Wang J, Yu H, Wei X, Yang C, Deng B. Power spectral density and coherence analysis of Alzheimer’s EEG. Cogn Neurodyn. 2015;9(3):291–304. 25972978 10.1007/s11571-014-9325-x PMC4427585

[45] Torres-Lista V, De la Fuente M, Giménez-Llort L. Survival Curves and Behavioral Profiles of Female 3xTg-AD Mice Surviving to 18-Months of Age as Compared to Mice with Normal Aging. J Alzheimers Dis Rep. 2017;1(1):47–57. 30480229 10.3233/ADR-170011 PMC6159713

[46] Yang L, Yan Y, Wang Y, Hu X, Lu J, Chan P, et al. Gradual Disturbances of the Amplitude of Low-Frequency Fluctuations (ALFF) and Fractional ALFF in Alzheimer Spectrum. Front Neurosci. 2018;12:975. 30618593 10.3389/fnins.2018.00975 PMC6306691

[47] Blackmore DG, Turpin F, Palliyaguru T, Evans HT, Chicoteau A, Lee W, et al. Low-intensity ultrasound restores long-term potentiation and memory in senescent mice through pleiotropic mechanisms including NMDAR signaling. Mol Psychiatry. 2021;26(11):6975–91. 34040151 10.1038/s41380-021-01129-7 PMC8760044

[48] Palop JJ, Mucke L. Amyloid-β–induced neuronal dysfunction in Alzheimer’s disease: from synapses toward neural networks. Nat Neurosci. 2010;13(7):812–8. 20581818 10.1038/nn.2583 PMC3072750

[49] Palop JJ, Mucke L. Network abnormalities and interneuron dysfunction in Alzheimer disease. Nat Rev Neurosci. 2016;17(12):777–92. 27829687 10.1038/nrn.2016.141 PMC8162106

[50] Mesik L, Parkins S, Severin D, Grier BD, Ewall G, Kotha S et al. Transcranial Low-Intensity Focused Ultrasound Stimulation of the Visual Thalamus Produces Long-Term Depression of Thalamocortical Synapses in the Adult Visual Cortex. J Neurosci. 2024;44(11):e0784232024. 38316559 10.1523/JNEUROSCI.0784-23.2024 PMC10941064

[51] Mitra A, Snyder AZ, Tagliazucchi E, Laufs H, Raichle ME. Propagated infra-slow intrinsic brain activity reorganizes across wake and slow wave sleep. eLife. 2015;4:e10781. 26551562 10.7554/eLife.10781 PMC4737658

[52] Meyer-Baese L, Jaeger D, Keilholz S. Neurovascular coupling: a review of spontaneous neocortical dynamics linking neuronal activity to hemodynamics and what we have learned from the rodent brain. J Neurophysiol. 2025;133(2):644–60. 39819035 10.1152/jn.00418.2024 PMC12175746

[53] Fultz NE, Bonmassar G, Setsompop K, Stickgold RA, Rosen BR, Polimeni JR, et al. Coupled electrophysiological, hemodynamic, and cerebrospinal fluid oscillations in human sleep. Science. 2019;366(6465):628–31. 31672896 10.1126/science.aax5440 PMC7309589

[54] Reddy PH. Abnormal tau, mitochondrial dysfunction, impaired axonal transport of mitochondria, and synaptic deprivation in Alzheimer’s disease. Brain Res. 2011;1415:136–48. 21872849 10.1016/j.brainres.2011.07.052 PMC3176990

[55] Castano-Prat P, Perez-Mendez L, Perez-Zabalza M, Sanfeliu C, Giménez-Llort L, Sanchez-Vives MV. Altered slow (< 1 Hz) and fast (beta and gamma) neocortical oscillations in the 3xTg-AD mouse model of Alzheimer’s disease under anesthesia. Neurobiol Aging. 2019;79:142–51. 31103943 10.1016/j.neurobiolaging.2019.02.009

[56] Quintana DD, Anantula Y, Garcia JA, Engler-Chiurazzi EB, Sarkar SN, Corbin DR, et al. Microvascular degeneration occurs before plaque onset and progresses with age in 3xTg AD mice. Neurobiol Aging. 2021;105:115–28. 34062487 10.1016/j.neurobiolaging.2021.04.019 PMC9703920

[57] Kubanek J, Shi J, Marsh J, Chen D, Deng C, Cui J. Ultrasound modulates ion channel currents. Sci Rep. 2016;6(1):24170. 27112990 10.1038/srep24170 PMC4845013

[58] Yoo S, Mittelstein DR, Hurt RC, Lacroix J, Shapiro MG. Focused ultrasound excites cortical neurons via mechanosensitive calcium accumulation and ion channel amplification. Nat Commun. 2022;13(1):493. 35078979 10.1038/s41467-022-28040-1 PMC8789820

[59] Chen J, Wang X, Li X, Li X, Zhang Y, Yuan Y. Ultrasound-Induced Synchronized Neural Activities at 40 Hz and 200 Hz Entrained Corresponded Oscillations and Improve Alzheimer’s Disease Memory. CNS Neurosci Ther. 2025;31(4):e70351. 40202152 10.1111/cns.70351 PMC11979792

[60] Song H, Chen R, Ren L, Zeng Y, Sun J, Tong S. Low intensity transcranial ultrasound stimulation induces hemodynamic responses through neurovascular coupling. iScience. 2024;27(7):110269. 39055926 10.1016/j.isci.2024.110269 PMC11269307

[61] Shen YY, Jethe JV, Reid AP, Hehir J, Amaral MM, Ren C, et al. Label free, capillary-scale blood flow mapping in vivo reveals that low-intensity focused ultrasound evokes persistent dilation in cortical microvasculature. Commun Biology. 2025;8(1):12. 10.1038/s42003-024-07356-2 PMC11704147 39762513

[62] Leinenga G, Götz J. Scanning ultrasound removes amyloid-β and restores memory in an Alzheimer’s disease mouse model. Sci Transl Med. 2015;7(278):278ra33. 25761889 10.1126/scitranslmed.aaa2512

[63] Chan D, Suk HJ, Jackson BL, Milman NP, Stark D, Klerman EB, et al. Gamma frequency sensory stimulation in mild probable Alzheimer’s dementia patients: Results of feasibility and pilot studies. PLoS ONE. 2022;17(12):e0278412. 36454969 10.1371/journal.pone.0278412 PMC9714926

[64] Matt E, Mitterwallner M, Radjenovic S, Grigoryeva D, Weber A, Stögmann E, et al. Ultrasound Neuromodulation With Transcranial Pulse Stimulation in Alzheimer Disease: A Randomized Clinical Trial. JAMA Netw Open. 2025;8(2):e2459170. 40009384 10.1001/jamanetworkopen.2024.59170 PMC11866033

[65] Chen X, You J, Ma H, Zhou M, Huang C. Transcranial pulse stimulation in Alzheimer’s disease. CNS Neurosci Ther. 2024;30(2):e14372. 37469252 10.1111/cns.14372 PMC10848065

[66] Dennison JL, Ricciardi NR, Lohse I, Volmar CH, Wahlestedt C. Sexual Dimorphism in the 3xTg-AD Mouse Model and Its Impact on Pre-Clinical Research. J Alzheimers Dis. 2021;80(1):41–52. 33459720 10.3233/JAD-201014 PMC8075398

