# Supplementary material for: Transcranial pulse stimulation modulates spectral signatures of Alzheimer’s disease in the 3×Tg-AD mouse model
Source: Alzheimers Res Ther. 2026 Jun 16;18:190. doi: 10.1186/s13195-026-02109-1 (PMC13501662; doi:10.1186/s13195-026-02109-1)
Supplement: Supplementary file 1 [file 13195_2026_2109_MOESM1_ESM.docx]

**Supplementary Information**

**Transcranial pulse stimulation modulates spectral signatures of Alzheimer’s disease in the 3×Tg-AD mouse model**

Irmak Gezginer^1,2,†^, Maria Eleni Karakatsani^1,2,†^, Prakruti Nanda^3^, Pranati Chalasani^3^, Diana Kindler^2^, Rafael Storz^4^, Markus Belau^4^, Ruiqing Ni^1,2,5^, Gerhard Schratt^3^, Xosé Luís Deán-Ben^1,2,*^, and Daniel Razansky^1,2,*^

^1^ Institute for Biomedical Engineering and Institute of Pharmacology and Toxicology, Faculty of Medicine, University of Zurich, Zurich, Switzerland

^2^ Institute for Biomedical Engineering, Department of Information Technology and Electrical Engineering, ETH Zurich, Zurich, Switzerland

^3^ Laboratory of Systems Neuroscience, Institute for Neuroscience, Department of Health Science and Technology, ETH Zürich, Zurich, Switzerland

^4^ Storz Medical AG, Tägerwilen, Switzerland

^5^ Department of Nuclear Medicine, Inselspital University of Bern, Bern, Switzerland

^†^ These authors have contributed equally

^*^Correspondence to: xl.deanben@pharma.uzh.ch or [daniel.razansky@uzh.ch](mailto:daniel.razansky@uzh.ch)

**Supplementary Figure 1.** Whole-brain functional connectivity (FC; n = 8 per group) matrices of wild-type (A) and 3xTg-AD (B) animals. ROIs are grouped and color-coded by functional network.

**Supplementary Figure 2.** Functional connectivity (FC) is lowered in 3×Tg-AD between caudoputamen and dentate gyrus. Data are shown as mean ± s.e.m.; two-tailed t-test, false-discovery-rate (FDR) < 0.05.

 **Supplementary Figure 3.** Inter-hemispheric connectivity of the endopiriform nucleus. Resting-state functional connectivity (FC, mean ± s.e.m.) between left and right endopiriform nuclei in WT and 3×Tg-AD mice. Two-tailed t-test, false-discovery-rate (FDR) < 0.05.

**Supplementary Figure 4.** Exploratory sex-stratified analysis of acute TPS effects in 3×Tg-AD mice. **A-B** Power-spectral density (PSD) of the low-frequency BOLD band (0.008–0.10 Hz) for each ROI under four conditions: WT, 3×Tg-AD before stimulation (AD-Pre), after one TPS train (AD-TPS1), and after a second TPS train (AD-TPS2). Shaded areas indicate s.e.m. Data for 3×Tg-AD mice are shown separately for males (**A**; n = 3) and females (**B**; n = 3), whereas WT data are shown as a common reference group. **C-D** Variance in the BOLD time series for the same ROIs, shown separately for male (**C**; n = 3) and female (**D**; n = 3) 3×Tg-AD mice. Bars indicate mean and error bars indicate s.e.m.; WT data are shown as a common reference group. Cg, anterior cingulate area dorsal part; Ins, agranular insular area dorsal part; Rs, retrosplenial area dorsal part; DG, dentate gyrus; Sub, subiculum; EP, endopiriform nucleus; CPu, caudoputamen; Hypo, lateral hypothalamic area; Th, posterior complex of the thalamus.

**Supplementary Figure 5.** Independent component analysis (ICA)-derived networks. Spatial maps of the independent components not shown in Fig. 4a. ICA identified 15 components, of which 13 correspond to biologically meaningful networks spanning the mouse brain.

**Supplementary Figure 6.** Power-spectral density (PSD) of the low-frequency BOLD band (0.008–0.10 Hz) for each ROI under the fixed-intensity paradigm. Each session began with a baseline resting-state fMRI scan (Pre) followed by sham handling. Mice then received four consecutive TPS trains, each followed by a resting-state fMRI acquisition (TPS1–TPS4). Shaded areas, s.e.m. Cg, anterior cingulate area dorsal part; Ins, agranular insular area dorsal part; Rs, retrosplenial area dorsal part; DG, dentate gyrus; EP, endopiriform nucleus; CPu, caudoputamen.

**Supplementary Figure 7.** Longitudinal TPS increases low-frequency BOLD power at 24 h and 120 h post-stimulation. Group-averaged power spectral density (PSD) pooled across all ROIs at baseline, 24 h post-TPS, and 120 h post-TPS (mean ± s.e.m., N = 5). Relative to baseline, PSD increased at 24 h and showed a further increase at 120 h. Stars denote frequency bins significantly different from baseline (blue, baseline vs 120 h; paired *t*-test with FDR correction, q < 0.05).

**Supplementary Figure 8.** Longitudinal TPS alters low-frequency BOLD spectra in hippocampal and entorhinal ROIs. ROI power spectral density (PSD) within the low-frequency BOLD band (0.008–0.10 Hz) at baseline, 24 h post-TPS, and 120 h post-TPS (mean ± s.e.m., N = 5) for hippocampal subfields (CA1, CA2) and the entorhinal area. Stars mark frequency bins that differ significantly from baseline (green, baseline vs 24 h; blue, baseline vs 120 h; paired *t*-test with FDR correction: *q* < 0.05 (*), *q* < 0.01 (**)).

**Supplementary Table 1.** List of regions used for analysis, based on Allen mouse brain atlas.

| **Region Name** | **Region Name (continued)** |
| --- | --- |
| Primary motor area | Field CA1 |
| Secondary motor area | Field CA2 |
| Primary somatosensory area, nose | Field CA3 |
| Primary somatosensory area, barrel field | Dentate gyrus |
| Primary somatosensory area, lower limb | Entorhinal area, lateral part |
| Primary somatosensory area, mouth | Entorhinal area, medial part |
| Primary somatosensory area, upper limb | Subiculum |
| Primary somatosensory area, trunk | Endopiriform nucleus |
| Supplemental somatosensory area | Lateral amygdalar nucleus |
| Gustatory areas | Striatum |
| Visceral area | Caudoputamen |
| Dorsal auditory area | Nucleus accumbens |
| Primary auditory area | Lateral septal nucleus |
| Posterior auditory area | Central amygdalar nucleus |
| Ventral auditory area | Globus pallidus |
| Anterolateral visual area | Bed nuclei of the stria terminalis |
| Anteromedial visual area | Thalamus |
| Lateral visual area | Ventral posterior complex of the thalamus |
| Primary visual area | Lateral group of the dorsal thalamus |
| Posterolateral visual area | Anterior group of the dorsal thalamus |
| Posteromedial visual area | Medial group of the dorsal thalamus |
| Postrhinal area | Hypothalamus |
| Anterior cingulate area, dorsal part | Periventricular zone of hypothalamus |
| Anterior cingulate area, ventral part | Anterior hypothalamic area |
| Prelimbic area | Ventromedial hypothalamic nucleus |
| Infralimbic area | Posterior hypothalamic nucleus |
| Orbital area, lateral part | Lateral hypothalamic area |
| Orbital area, medial part | Midbrain |
| Orbital area, ventrolateral part | Superior colliculus, sensory related |
| Agranular insular area, dorsal part | Inferior colliculus |
| Agranular insular area, posterior part | Substantia nigra, reticular part |
| Agranular insular area, ventral part | Ventral tegmental area |
| Retrosplenial area, lateral agranular part | Midbrain reticular nucleus |
| Retrosplenial area, dorsal part | Anterior pretectal nucleus |
| Retrosplenial area, ventral part | Cuneiform nucleus |
| Anterior area | Red nucleus |
| Rostrolateral visual area | Pedunculopontine nucleus |
| Temporal association areas | Pons |
| Perirhinal area | Nucleus of the lateral lemniscus |
| Ectorhinal area | Principal sensory nucleus of the trigeminal |
| Anterior olfactory nucleus | Parabrachial nucleus |
| Piriform area | Pontine reticular nucleus |
| Cortical amygdalar area | Superior central nucleus raphe |
| Hippocampal formation |  |

**Supplementary Table 2.** Acoustic descriptors reported for the applied energy flux density of 0.05 mJ/mm² at PRF = 4 Hz, in free water and after murine skull derating.

| **Parameter** | **Free water**  **TPS** | **Derated**  **TPS** | **FDA / ITRUSST limit**  **FUS** | **Status** |
| --- | --- | --- | --- | --- |
| Spatial-Peak Temporal-Average Intensity (I_SPTA_, primary thermal safety metric) | 16 mW/cm² = 100 mW/cm²(max) × (0.05/0.25) × (4/5) | ~6.7 mW/cm² (× skull T² = 0.42) | 94 mW/cm² (FDA diagnostic cephalic) 720 mW/cm² (transcranial Doppler) | **14× below limit** |
| Spatial-Peak Pulse-Average Intensity (I_SPPA_, mechanical bioeffect metric) | 22 W/cm² = 111 W/cm²(max) × (0.05/0.25) | ~9.3 W/cm² (× skull T² = 0.42) | 190 W/cm² (FDA cephalic limit) | **20× below limit** |
| Peak rarefactional pressure (P_r_) (cavitation-relevant metric) | ~2.2 MPa (scaled from max *ex-vivo* measurement) | ~1.4 MPa (× skull T_pressure_ = 0.65) | No specific limit for TPS;  MI N/A for TPS single pulses | **Well below tissue damage threshold (40 MPa)** |
| Pulse duration | ~3 µs (single broadband pulse) | ~3 µs (unchanged) | — | **N/A — TPS-specific** |
| Duty cycle | 3 µs × 4 Hz = 0.0012% | 0.0012% | — | **Extremely low** |
| Center / dominant frequency | Broadband ~0.1–3 MHz; dominant energy ~0.3–1 MHz. No sinusoidal carrier. | Same spectrum | 220–650 kHz typical for sinusoidal FUS | **N/A — broadband pulse, no single carrier frequency** |
| Mechanical index (MI) | N/A — MI formulation assumes sinusoidal waveform dynamics | Not applicable | MI ≤ 1.9 (for sinusoidal TUS only) | **N/A** |
| EFD (TPS-native dosimetry metric) | 0.05 mJ/mm² (20% of CE-approved max) | ~0.021 mJ/mm² | ≤ 0.25 mJ/mm² (CE-approved clinical maximum) | **Well within CE limits** |
